# Cost-effectiveness of early versus delayed disease-modifying antirheumatic drugs for psoriatic arthritis

**DOI:** 10.1016/j.lana.2026.101661

**Published:** 2026-10-14

**Authors:** Sabrina Hundal, Julian Cappelli, Savanah Haidar, Mohamed Osman, Mohammed Kaouache, Elham Rahme, Stephanie R. Goldberg, Dafna D. Gladman, Julien Ringuet, Anastasiya Muntyanu, Alexander Tsoukas, Elena Netchiporouk

**Affiliations:** a Department of Medicine, University of Calgary, Calgary, Alberta, Canada; b Division of Experimental Medicine, McGill University Health Centre, Montreal, Quebec, Canada; c Department of Health Research Methods, McMaster University, Hamilton, Ontario, Canada; d Department of Medicine, University of Montreal, Montreal, Quebec, Canada; e Department of Rheumatology, University of Alberta, Edmonton, Alberta, Canada; f Department of Mathematics and General Sciences, Prince Sultan University, Riyadh, Saudi Arabia; g Division of Clinical Epidemiology, McGill University, Montreal, Quebec, Canada; h Department of Surgery, Mary Washington Healthcare, Fredericksburg, VA, USA; i Division of Rheumatology, University of Toronto, Toronto, Ontario, Canada; j Schroeder Arthritis Institute, Toronto Western Hospital, University Health Network, Toronto, Ontario, Canada; k Centre de Recherche Dermatologique du Québec (CRDQ), Quebec, Canada; l Division of Dermatology, McGill University Health Centre, Montreal, Quebec, Canada; m Division of Dermatology, Department of Medicine, University of Toronto, Toronto, Ontario, Canada; n Division of Rheumatology, McGill University Health Centre, Montreal, Quebec, Canada

**Keywords:** Psoriatic arthritis, DMARD, Economic evaluation, Cost-utility analysis, Minimal disease activity, Quality-adjusted life-years, Markov model, Value-based care

## Abstract

**Background:**

Disease-modifying antirheumatic drugs (DMARDs) are standard first-line therapy for psoriatic arthritis (PsA). Observational studies suggest earlier initiation is associated with superior minimal disease activity (MDA) attainment, yet the economic and health implications of treatment timing remain uncertain. This cost-utility analysis evaluated the cost-effectiveness of early versus delayed DMARD initiation in DMARD-naive adults with PsA from a U.S. payer perspective.

**Methods:**

We built a cohort state-transition (Markov) model (monthly cycles, 15-year horizon) from published data (2000–2025). Patients initiated a DMARD under two strategies: early (≤1 year post-diagnosis) and delayed initiation (>1 year). Patients remained in the pre-treatment state for six months prior to response assignment, then transitioned among complete response (sustained MDA), partial response (non-sustained MDA), non-response, no treatment, and death. Primary outcomes included the incremental cost-effectiveness ratio (ICER) and incremental net monetary benefit (INMB). Uncertainty intervals (UIs) were derived from probabilistic sensitivity analyses.

**Findings:**

Early DMARD initiation provided an additional 0.56 (95% UI, 0.32–0.83) QALYs at an incremental cost of –US$43,902.94 (95% UI, –US$147,096.29 to US$61,615.86) per patient relative to delayed initiation, yielding a favourable ICER (–US$78,398.11/QALY). At a willingness-to-pay threshold of US$50,000/QALY, mean INMB was US$71,902.94 (95% UI, –US$36,663.51 to US$176,658.91), with early initiation preferred in 90.6% of simulations.

**Interpretation:**

Early DMARD initiation was associated with improved long-term health outcomes at reduced costs, suggesting timely DMARD initiation may represent a high-value strategy. Given the observational basis of the key treatment-timing input, findings support disease-duration stratified analyses of existing phase III trials and further characterization of access delays.

**Funding:**

None.

**Research in context**

**Evidence before this study**

Psoriatic arthritis (PsA) is a chronic inflammatory disease associated with progressive structural joint damage, functional impairment, and reduced health-related quality of life. Observational cohort studies consistently demonstrate that delays in diagnosis and initiation of disease-modifying antirheumatic drugs (DMARDs) are associated with worse long-term outcomes, including irreversible joint damage, persistent functional disability, and a lower likelihood of attaining minimal disease activity or sustained remission. Existing economic evaluations in PsA have largely focused on the cost-effectiveness of individual pharmacologic agents or treatment sequences, with limited consideration of treatment timing or the long-term economic consequences of delayed disease control on health outcomes and healthcare resource use. We searched MEDLINE from Jan 1, 2000 to Dec 15, 2025, for English-language studies using terms for psoriatic arthritis, antirheumatic agents, minimal disease activity, health utilities, economic evaluation, healthcare resource utilization, and costs. No published studies were identified that evaluated the cost-effectiveness of early versus delayed initiation of DMARD therapy in adults with PsA.

**Added value of this study**

This study provides the first cost-utility analysis comparing early versus delayed initiation of DMARD therapy in DMARD-naive adults with PsA. By integrating longitudinal clinical outcomes, EQ-5D utilities, and U.S. healthcare costs over a 15-year horizon within a unified framework, this analysis quantifies the potential economic value of early disease control beyond drug-specific comparisons.

**Implications of all the available evidence**

Across immune-mediated inflammatory diseases, converging translational, observational, and trial evidence supports the clinical plausibility of earlier disease-modifying therapy improving long-term outcomes. If the observed association between earlier DMARD initiation and improved disease control reflects a true treatment-timing effect in PsA, earlier DMARD initiation may improve long-term health outcomes while reducing overall healthcare costs compared with delayed treatment. These findings identify timely DMARD initiation as a potentially high-value strategy and an important priority for confirmation through stratified analyses of existing phase III trial data, with implications for clinical practice, guideline development, and reimbursement frameworks that may restrict access to timely DMARD therapy.

## Introduction

Psoriatic arthritis (PsA) is a chronic immune-mediated inflammatory musculoskeletal disease affecting 25–40% of patients with psoriasis, a chronic inflammatory skin disorder.^1^ PsA leads to progressive joint damage and substantial impairment in physical function and quality of life.^1^ In addition to peripheral arthritis, many patients develop axial disease, enthesitis, dactylitis, and cutaneous involvement, contributing to a heterogeneous clinical course and a high cumulative disease burden.^1^ Observational cohort studies have consistently shown that delays in diagnosis and treatment are associated with irreversible structural damage, persistent functional disability, and reduced likelihood of attaining sustained disease control.^2^^,^^3^

These observations have been interpreted as supporting a therapeutic “window of opportunity” in PsA, analogous to that described in rheumatoid arthritis, during which timely intervention may alter disease trajectory.^4^ Translational studies provide a biological rationale for this hypothesis: the transition from psoriasis to PsA involves distinct T-cell alterations, including tissue-resident memory CD8+ T-cell signatures and clonal expansion of activated, tissue-homing synovial CD8+ T cells, suggesting that earlier intervention may interrupt self-sustaining inflammation before it becomes immunologically entrenched.^5^ This rationale is supported by observational data from multiple independent PsA registries linking diagnostic or treatment delays with poorer outcomes, as well as emerging randomized evidence from early intensive treatment strategies favouring earlier therapy.^2^^,^^3^^,^^6^^,^^7^ Nevertheless, the window-of-opportunity hypothesis in PsA remains predominantly informed by observational comparisons across diagnostic or treatment-delay groups, which are vulnerable to confounding by indication, differences in referral pathways, access to care, disease phenotype, and treatment selection.

Despite this rationale, treatment initiation in PsA is frequently delayed. Delays have been linked to the heterogeneous and often subtle clinical presentation of PsA, absence of a diagnostic biomarker, delays in symptom recognition and rheumatology referral, diagnostic uncertainty, and barriers to accessing effective therapy after diagnosis.^3^^,^^4^ Even after diagnosis, escalation to disease-modifying antirheumatic drugs (DMARDs) is often conservative, with prolonged reliance on nonsteroidal anti-inflammatory drugs or stepwise conventional synthetic DMARD (csDMARDs) therapy.^8^ This stepwise approach often requires failure of conventional DMARDs before initiating more effective therapies, such as biologic DMARDs (bDMARDs).^8^ Over the past decade, multiple bDMARDs and targeted synthetic DMARDs (tsDMARDs) have demonstrated efficacy across PsA domains, expanding therapeutic options but also increasing treatment complexity and costs. While clinical guidelines increasingly emphasize early and effective disease control, reimbursement and access policies in many jurisdictions continue to favour delayed initiation of advanced therapies, reflecting concerns regarding long-term cost and uncertainty about comparative value across treatment strategies.^8^

Economic evaluations in PsA have predominantly examined the cost-effectiveness of individual DMARDs relative to placebo or standard care, typically in established disease. However, these analyses do not address a central question facing clinicians and policymakers: whether earlier initiation of DMARD therapy, as opposed to delayed escalation, confers greater long-term value by improving health outcomes and reducing downstream healthcare utilization. Moreover, few studies have evaluated treatment timing across multiple available therapies within a unified analytic framework, limiting the applicability of existing evidence to real-world treatment pathways. To address this gap, we conducted a cost-utility analysis comparing early versus delayed DMARD initiation in DMARD-naive adults with PsA, incorporating all DMARDs with sufficient evidence for economic modelling.

## Methods

### Study design

We conducted a cost-utility analysis comparing early versus delayed DMARDs in DMARD-naive adults with PsA. The analysis was performed from the perspective of the U.S. healthcare system and followed the Consolidated Health Economic Evaluation Reporting Standards.^9^ A glossary of commonly used cost-utility analysis terms can be found in Supplementary Table S1. As this study used aggregate data from published sources, institutional review board approval and patient consent were not required. The model was a cohort state-transition Markov model parameterized from published summary statistics; it estimated the proportion of a hypothetical cohort occupying each health state over time and did not generate synthetic patient-level data.

### Data sources and input parameters

A targeted literature review (TLR) of MEDLINE (2000–2025) conducted by two independent reviewers (SH, JC) identified studies for five domains: clinical outcomes, EuroQol 5-Dimension (EQ-5D) utilities, mortality, resource use and costs, and economic evaluations. Inclusion criteria, PICOS framework, and search strategy are in Supplementary Tables S2 and S3.

### Target population and intervention

The modelled population comprised adults with PsA diagnosed by a rheumatologist, consistent with Classification Criteria for Psoriatic Arthritis (CASPAR)–defined cohorts reported in the literature.^10^ Patients were assumed to be DMARD-naive at diagnosis, reflecting contemporary early-disease cohorts. Accordingly, we evaluated the timing of first DMARD initiation among patients for whom systemic therapy was clinically indicated. Patients with milder disease managed with nonsteroidal anti-inflammatory drugs alone were outside the modelled population and decision problem. Baseline demographic characteristics (age, sex distribution) and functional status were informed by large observational PsA studies and registries.

Early DMARD initiation was defined as treatment initiation within 12 months of PsA diagnosis, reflecting common thresholds used in cohort studies evaluating treatment delay.^3^^,^^4^^,^^11^ Delayed initiation was defined as DMARD initiation more than 12 months after diagnosis, with mean delay parameters informed by real-world registry data. This comparator was intended to represent treatment delays observed in routine care, rather than deliberate withholding of indicated DMARD therapy.

Disease burden was incorporated through minimal disease activity (MDA)-defined response states, EQ-5D utilities stratified by response state, and Health Assessment Questionnaire Disability Index (HAQ-DI)–linked medical cost adjustments. Formal stratification by baseline disease severity was not undertaken because severity-specific transition probabilities, utilities, and costs were not consistently available across included therapies.

### Interventions and comparators

The analysis incorporated all DMARDs with sufficient evidence for economic modelling. Therapies evaluated included csDMARDs (methotrexate), tsDMARDs (apremilast and tofacitinib), and bDMARDs (adalimumab, etanercept, certolizumab pegol, golimumab, infliximab, ixekizumab, secukinumab, ustekinumab, guselkumab, and bimekizumab). Full details of included therapies and dosing are provided in Supplementary Table S4.

Drug acquisition costs accrued by cycle only while patients remained on a given agent. Patients could remain on therapy, switch, discontinue, enter the no treatment state, or restart therapy according to modelled transition probabilities. Thus, the 15-year horizon reflected long-term disease management with treatment attrition and sequencing, rather than continuous lifelong exposure to the initial DMARD.

The DMARD-class distribution at model entry for the base case was taken directly from the source registry for each cohort and therefore differs modestly between strategies (early: csDMARD 17.5%, tsDMARD 24.0%, bDMARD 58.5%; delayed: csDMARD 19.4%, tsDMARD 12.9%, bDMARD 67.7%), reflecting real-world prescribing.^3^ Combination csDMARD therapy was not modelled as a distinct regimen because long-term, regimen-specific data for individual combinations were unavailable.

### Model structure and health states

A state-transition Markov model in TreeAge Pro Healthcare 2025 (TreeAge LLC) simulated the disease course of PsA using monthly cycles over a 15-year time horizon, selected to reflect the chronic course of PsA while minimizing speculative lifetime extrapolation. The model comprised six mutually exclusive health states: pre-treatment, complete response, partial response, non-response, no treatment and death (absorbing state). Response states applied to both the first-line and second-line (post-switch) therapy. A schematic of the model structure is shown in Fig. 1.

**Fig. 1:**
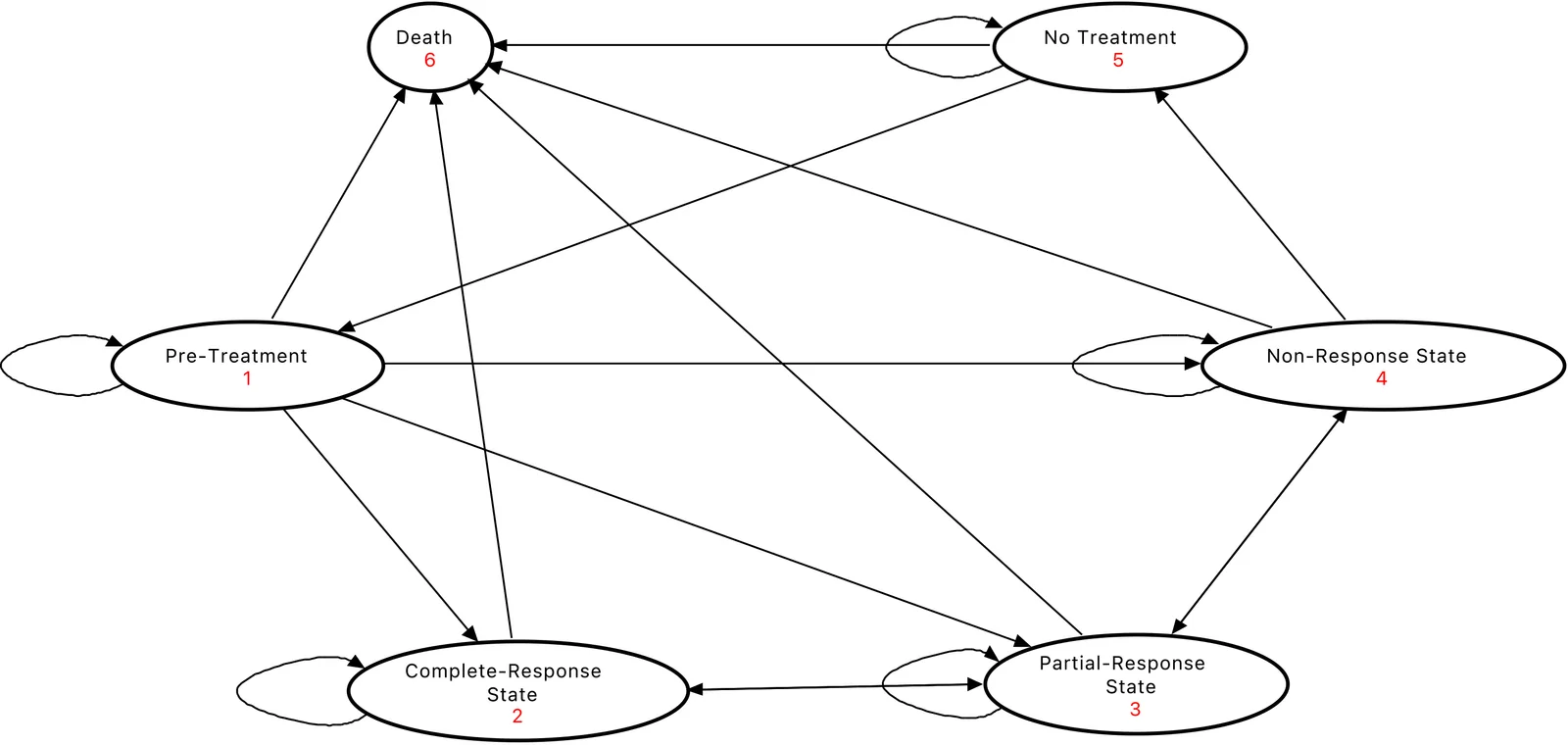
Markov model state–transition diagram. Diagram showing the six health states and allowable transitions in the Markov model evaluating early versus delayed disease-modifying antirheumatic drug initiation in psoriatic arthritis. Arrows indicate possible monthly transitions, with death as an absorbing state. Patients remain in pre-treatment for six monthly cycles before initial response assignment. Response states apply to both first-line and second-line (post-switch) therapy.

Response states were defined according to MDA status, which is a recommended PsA treatment target and has well-established associations with long-term outcomes.^12^^,^^13^ Complete response was defined as meeting MDA criteria (defined in Supplementary Table S5) in the current and preceding cycle. Partial response was defined as meeting MDA criteria in the current cycle but not the preceding cycle. Non-response was defined as not meeting MDA criteria.

All patients entered the pre-treatment state for six monthly cycles, representing the initial response-assessment interval before response status was assigned. Patients were then allocated to complete response, partial response, or non-response at six months, consistent with MDA assessment time points used in PsA trials and registries. The one-month cycle length was used for model updating and accrual of costs and QALYs. After initial response assignment, subsequent movement between response states was governed by monthly transition probabilities derived from longitudinal MDA data. Sensitivity to the response-assessment window was examined using 3- and 12-month alternatives.

Transitions followed a bidirectional stepwise structure in which the complete-response state could transition to the partial-response state, and the partial-response state could transition to the non-response state, with each transition also permitted in reverse. Loss of response was therefore modelled over consecutive cycles rather than as an abrupt complete response to non-response transition within a single cycle, which was not supported by the available longitudinal data and was considered less clinically plausible. After response assessment, individuals in the non-response state could remain in non-response or discontinue to the no treatment state. Patients in the no treatment state could remain off treatment or switch to second-line DMARD therapy and enter second-line response states, using observed real-world transition probabilities.^14^ Discontinuation and switching were applied only after response assessment as the pre-treatment state represented the initial assessment interval rather than a long-term treatment state. Switchers initiated a second DMARD with response probabilities adjusted for prior treatment exposure.^15^^,^^16^ Full model parameters are provided in Supplementary Table S5.

Adverse events were not modelled as separate transient health states. Their effects were captured indirectly through discontinuation and switching pathways, which included treatment-emergent adverse events as reasons for cessation, and through higher secondary costs assigned to discontinuers and switchers. Separate adverse-event disutilities and management costs were not added because event-specific estimates were unavailable across included DMARD classes and could have double-counted effects already captured in these pathways.

### Transition probabilities

Differences between early versus delayed cohorts were derived from cohort and registry data of DMARD treatment in DMARD-naive adults with PsA stratified by treatment timing.^3^^,^^4^^,^^11^ The 6-month early versus delayed MDA relative risk (1.88; 95% confidence interval 1.12–3.18) was derived from a U.S. registry to inform initial response stratification and subsequently applied multiplicatively to drug-specific MDA probabilities estimated from randomized trials and longitudinal studies.^3^

To limit bias from heterogeneous follow-up durations across therapies, drug-specific MDA probabilities were estimated over a common 12-month response window, corresponding to the minimum follow-up available across included drugs. Among patients meeting MDA criteria, assignment to complete versus partial response was informed by the observed distribution of sustained versus non-sustained MDA in longitudinal cohort data.^11^ Month 12 transition probabilities were held constant through month 36, the longest period with delay-stratified comparative longitudinal data.

From cycle 36 onward, transitions from non-response to response were multiplicatively attenuated and transitions from response to non-response increased using exponential functions with common decay parameters (α = β = 0.01). This conservative approach, a standard practice in economic models to extrapolate beyond observed data, was not intended to represent true biologic response decay but rather to limit bias from unequal follow-up while reflecting uncertainty in long-term treatment durability.^17^ Drug-specific transition probabilities were retained for each therapy and summarized in Supplementary Table S5.

### Mortality

All-cause mortality used U.S. life-table probabilities, adjusted by age- and sex-specific standardized mortality ratios for North American PsA, with additional adjustment to account for DMARD exposure.^18^^,^^19^ Face validity was assessed by comparing model-predicted mortality with published U.S. epidemiologic data.^20^

### Health utilities

Utilities were applied by health state using EQ-5D estimates (baseline 0.59; partial-response and non-response decrements of −0.08 and −0.24 relative to complete response), with an early-cohort utility multiplier (base case 1.0) examined only in scenario analysis.^11^

### Costs and resource use

Costs were reported in 2025 US dollars, discounted at 3% annually, and inflated using the medical component of the Consumer Price Index. Costs comprised primary index-DMARD acquisition costs and secondary DMARD-specific health care costs. Primary drug acquisition costs were based on January 2025 U.S. wholesale acquisition cost (WAC) data and accrued according to regimen-specific dosing while patients remained on the index DMARD (Supplementary Table S4). In agent-stratified analyses, the same per-cycle acquisition cost was applied to both arms for the specified drug. Total costs were then accumulated over monthly cycles according to treatment received, time on treatment, health-state occupancy, and associated secondary healthcare costs.

Secondary DMARD-specific medical costs were derived from U.S. claims-based estimates in treatment-naive PsA and applied separately from primary index-DMARD acquisition costs.^21^ These costs captured downstream health care resource use associated with each treatment pathway, including inpatient care, outpatient care, emergency department care, physician and other outpatient services, and non-index pharmacologic costs. In each monthly cycle, total cost was calculated as the WAC-based acquisition cost of the current index DMARD plus the corresponding secondary DMARD-specific health care cost, adjusted by treatment pathway, time, and health state. HAQ-DI–linked cost adjustments were applied to reflect differences in functional burden across response states.^22^ The composition and application of secondary costs are detailed in Supplementary Table S6.

### Outcome measures

Primary economic outcomes were the incremental cost-effectiveness ratio (ICER) and incremental net monetary benefit (INMB), using a willingness-to-pay (WTP) threshold of US$50,000 per quality-adjusted life-year (QALY). Base case costs and QALYs are deterministic mean per-patient estimates.

### Sensitivity analyses

Analyses were conducted in TreeAge Pro Healthcare 2025 and Excel (version 16.103.3). Model inputs were parameterized using summary statistics abstracted from the TLR, with reported means, standard deviations (SD), and confidence intervals used to derive distributions.

Uncertainty was explored using probabilistic sensitivity analyses with 10,000 Monte Carlo simulations, varying all parameters according to their prespecified distributions. Results were summarized using cost-effectiveness planes and acceptability curves. 95% uncertainty intervals (UIs) for incremental costs, QALYs, and INMB were calculated from the 2.5th and 97.5th percentiles of the probabilistic sensitivity analysis distributions and represent parameter uncertainty, not sampling variability from simulated individuals. Deterministic one-way sensitivity analyses were performed on key parameters.

Scenario analyses included a lifetime horizon, alternative discount rates (0%, 5%), and alternative response-assessment windows (3, 12 months). To assess whether results were driven by the observed treatment mix, we also repeated the analysis using an identical index-DMARD distribution in both strategies. To address uncertainty in U.S. drug acquisition costs, we modelled biologic acquisition-cost reductions of 15% and 60% from WAC and a drug-specific net-price scenario applying published 2019 list-to-net discounts for self-administered systemic psoriatic therapies, with class-based proxy discounts (mean discount for the same mechanism of action) for biologics not in that source and an established net-pricing estimate for Janus kinase (JAK) inhibitors.^23^, ^24^, ^25^

To address confounding by indication in the observational early versus delayed MDA estimate, we conducted a quantitative bias analysis. We computed an E-value for the base case MDA relative risk and its lower 95% confidence limit. We also conducted a standardized attenuation analysis in which initial response state probabilities were recalculated as the MDA relative risk was progressively attenuated from 1.88 toward the null (1.00), with all other inputs within the standardized model held fixed. To assess transportability, we conducted a stress-test scenario varying non-U.S.-derived clinical, utility, mortality, and treatment-pattern inputs by ±50% while holding U.S.-derived inputs fixed (Supplementary Table S7).

### Role of funding source

No funding.

## Results

### Targeted literature review

The TLR identified 93 studies (Supplementary Fig. S1). Of these, 24 studies informed transition probabilities for the base-case analysis, three studies provided health utility estimates, and six studies contributed cost data. No published economic evaluations directly comparing early versus delayed DMARD initiation in PsA were identified. Studies informing model inputs were published between 2000 and 2025. All model parameters, distributions, data sources, and key assumptions are summarized in Supplementary Table S5. Quality was appraised using ROBINS-I and RoB 2 (Supplementary Tables S8 and S9, respectively). The modelled cohort had a mean (SD) age of 53 (13) years at DMARD initiation, with 59% female patients in the early initiation strategy and 52% female patients in the delayed initiation strategy.

### Base case analysis

Over 15 years, early DMARD initiation accrued US$1,134,851.91 in direct medical costs and 8.62 QALYs per patient, compared with US$1,178,754.85 and 8.06 QALYs for delayed initiation. Early initiation provided an incremental gain of 0.56 QALYs (95% UI, 0.32 to 0.83) at an incremental cost of –US$43,902.94 per patient (95% UI, –US$147,096.29 to US$61,615.86). Hence, mean per-patient costs were $43,902.94 lower with early than with delayed initiation. Early initiation was therefore dominant (ICER –US$78,398.11/QALY), below the WTP threshold of US$50,000/QALY. Health state occupancy over time in the base case is shown in Supplementary Table S10.

### Class-stratified results

Table 1 presents class-stratified analyses in which each row compares early versus delayed initiation of the same DMARD class or agent, rather than head-to-head comparisons between therapies. Early initiation was associated with positive INMB across all evaluated therapies, although the magnitude varied.

**Table 1:** Incremental net monetary benefit and incremental cost-effectiveness ratio for early versus delayed initiation, by DMARD class and key scenario analyses.

Analysis	INMB (US$), WTP US$50,000/QALY	ICER (US$/QALY)
Base case, overall (95% UI)	71,902.94 (−36,663.51 to 176,658.91)	−78,398.11
csDMARD: methotrexate	161,449.26	−214,670.92
tsDMARD: apremilast	94,988.00	−100,774.60
tsDMARD: tofacitinib	86,609.60	−83,245.54
bDMARD: TNF inhibitors	49,606.33	−28,740.21
bDMARD: IL-12/23 inhibitor	73,205.90	−68,074.03
bDMARD: IL-23 inhibitor	10,747.47	30,808.09
bDMARD: IL-17 inhibitors	312.16	49,349.67
bDMARD: JAK inhibitor	21,399.03	19,860.52
Scenario Analyses		
Biologic acquisition cost reduction, 15% WAC	57,485.86	−52,653.32
Biologic acquisition-cost reduction, 60% WAC	65,140.24	−66,321.86
Drug-specific net-price scenario	93,738.47	−117,390.13
Identical index-DMARD distribution	94,600.75	−102,581.85
Transportability: non-U.S. inputs +50%	57,541.36	−36,541.36
Transportability: non-U.S. inputs −50%	74,786.27	−45,286.27
Time horizon, 1 year	3579.95	−128,997.50
Time horizon, lifetime	85,024.87	−68,810.04
Discount rate, 0%	88,134.65	−77,731.38
Discount rate, 5%	63,321.55	−79,227.65
Response-assessment window, 3 months	73,221.23	−78,458.30
Response-assessment window, 12 months	70,314.32	−85,219.85

Class- and agent-stratified rows compare early versus delayed initiation within the same DMARD class or agent and should not be interpreted as head-to-head comparisons between therapies. Scenario-analysis rows vary the specified model assumption relative to the base case.

ICER, incremental cost-effectiveness ratio; IL, interleukin; INMB, incremental net monetary benefit; JAK, Janus kinase; QALY, quality-adjusted life-year; TNF, tumour necrosis factor; UI, uncertainty interval; US$, United States Dollar; WAC, wholesale acquisition cost; WTP, willingness-to-pay.

Early initiation of methotrexate yielded the highest INMB (US$161,449.26) and was dominant relative to delayed initiation, with the high INMB reflecting the lowest WAC across DMARDs combined with downstream reduced secondary costs shared by all agents. Early initiation of tsDMARDs also produced favourable INMBs (apremilast: US$94,988.00; tofacitinib: US$86,609.60) and retained dominance.

Among bDMARDs, early initiation of tumour necrosis factor (TNF) inhibitors and interleukin (IL) 12/23 inhibitors yielded positive INMBs (US$49,606.33 and US$73,205.90, respectively) and was dominant. Comparatively, early initiation of IL-23, IL-17, and JAK inhibitors yielded smaller but positive INMBs (US$10,747.47, US$312.16, US$21,399.03 respectively) and positive ICERs (US$30,808.09, US$49,349.67, US$19,860.52 respectively), though all remaining below the WTP threshold of US$50,000/QALY.

### Probabilistic sensitivity analyses

Probabilistic sensitivity analyses demonstrated that early DMARD initiation was associated with higher QALYs and lower costs in the majority of simulations. The incremental cost-effectiveness plane (Fig. 2) shows that 80.5% (8045 of 10000) of simulations fell in the southeast quadrant, indicating dominance of early initiation. Few simulations (9.4%) crossed the WTP threshold line.

**Fig. 2:**
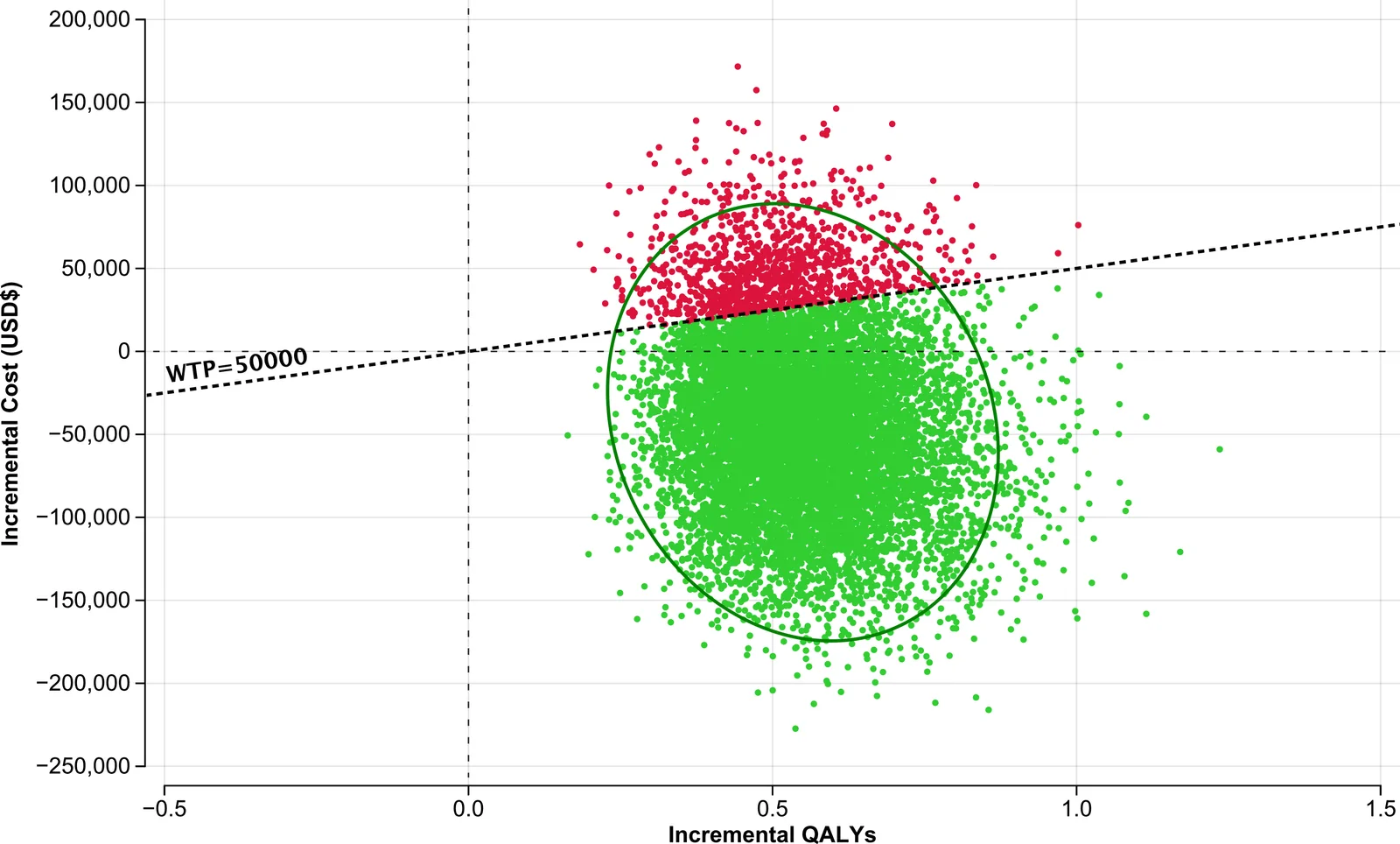
Incremental cost-effectiveness scatterplot. Each point represents a probabilistic iteration comparing early versus delayed disease-modifying antirheumatic drug initiation in psoriatic arthritis. The x-axis shows incremental QALYs gained with early initiation, and the y-axis shows incremental costs (2025 US dollars). The dashed vertical and horizontal lines represent no incremental difference in effectiveness and cost, respectively. The diagonal dashed line represents the willingness-to-pay (WTP) threshold of US$50,000/QALY. Points falling below this line indicate simulations in which early initiation is cost-effective relative to delayed initiation. Green points indicate simulations in which early initiation resulted in a higher net monetary benefit than delayed initiation, whereas red points indicate simulations in which delayed initiation was favoured. The ellipse represents the 95% uncertainty region of the joint distribution of incremental costs and QALYs. Abbreviations: QALY, quality-adjusted life-year; US$, United States Dollar; WTP, willingness-to-pay.

The cost-effectiveness acceptability curve (Fig. 3) showed that early initiation was the preferred strategy across the full range of WTP thresholds evaluated. At a WTP of US$50,000/QALY, early initiation was favoured in 90.6% of simulations, compared with 9.4% for delayed initiation. The corresponding mean INMB was US$71,902.94 (95% UI, –US$36,663.51 to US$176,658.91).

**Fig. 3:**
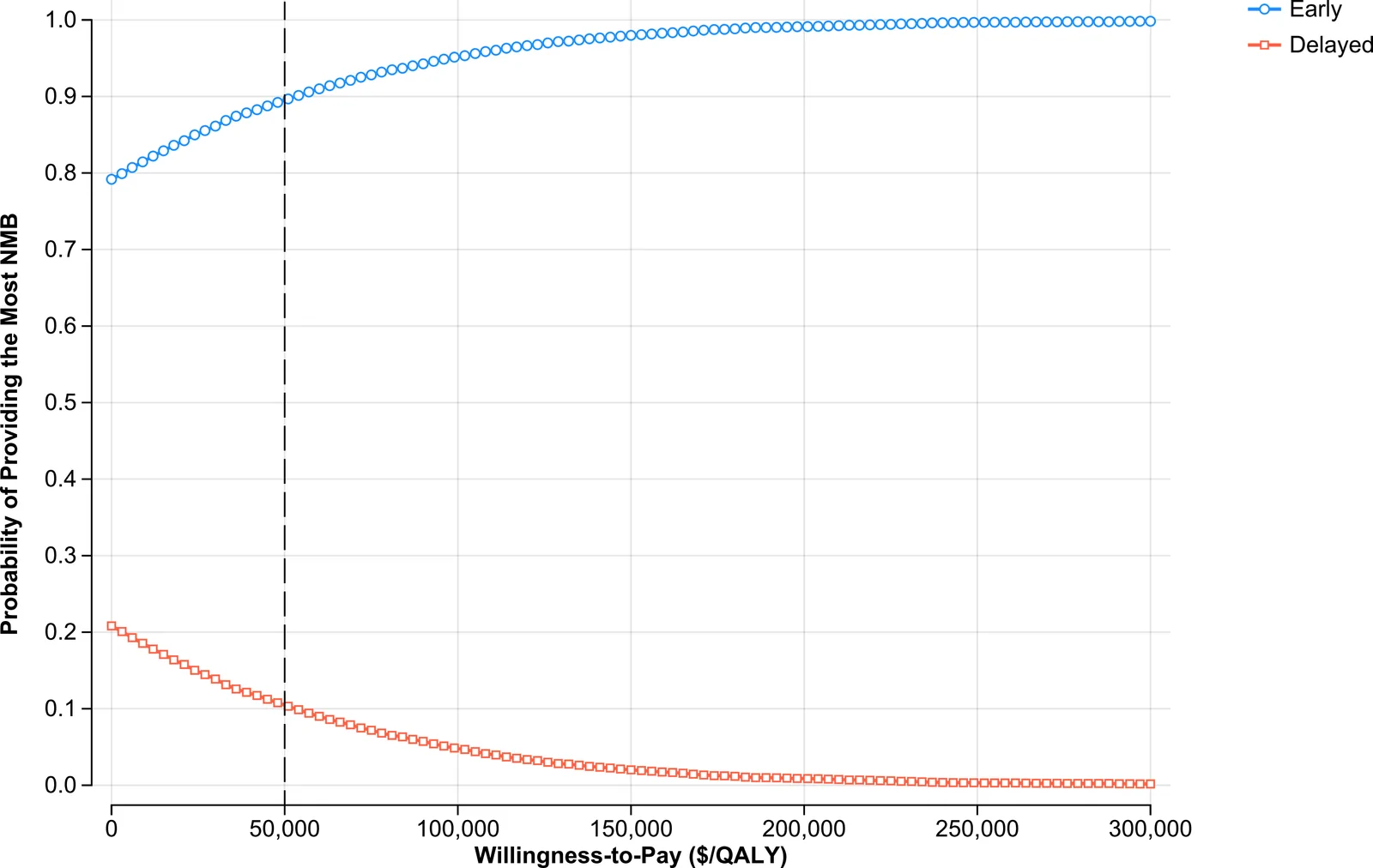
Cost-effectiveness acceptability curve. Cost-effectiveness acceptability curve showing the probability that early or delayed disease-modifying antirheumatic drug initiation is the most cost-effective strategy across willingness-to-pay thresholds, based on 10,000 probabilistic Monte Carlo simulations. Abbreviations: NMB, net monetary benefit; QALY, quality-adjusted life-year; US$, United States Dollar.

### Deterministic sensitivity analyses

The tornado diagram (Fig. 4) illustrates the impact of parameter uncertainty on the INMB at a WTP of US$50,000/QALY. Corresponding effects on total costs and QALYs are presented in Supplementary Figs. S2 and S3, respectively.

**Fig. 4:**
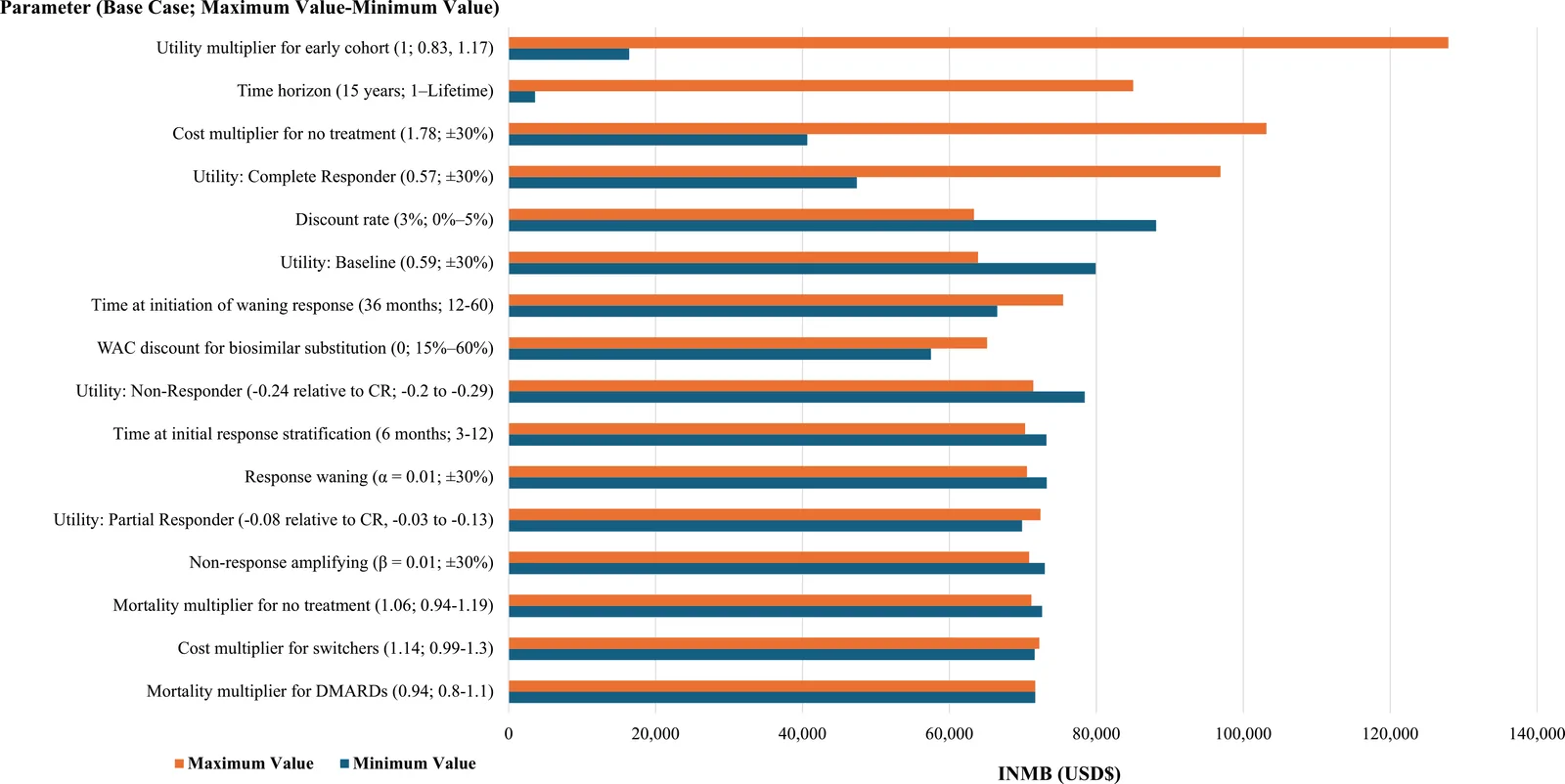
One-way sensitivity analysis of incremental net monetary benefit. Deterministic one-way sensitivity analysis showing the effect of varying key model parameters on the incremental net monetary benefit (INMB) of early versus delayed disease-modifying antirheumatic drug initiation. Parameters are listed on the vertical axis with their minimum and maximum values, and the horizontal axis reflects INMB. Positive values indicate that early initiation is more cost-effective at a willingness-to-pay threshold of US$50,000/QALY. Bars represent INMB when each parameter is set to its minimum (blue) or maximum (orange) value, and the dashed vertical line indicates the base case estimate. Abbreviations: INMB, incremental net monetary benefit; QALY, quality-adjusted life-year; US$, United States Dollar; WAC, wholesale acquisition cost.

The model was most sensitive to parameters governing health utility and time, specifically the utility multiplier applied to the early initiation cohort (INMB range, US$16,402.94 at a multiplier of 0.83 to US$127,902.94 at a multiplier of 1.17), the time horizon (INMB range, US$3579.95 at 1 year to US$85,024.87 at a lifetime horizon), and the cost multiplier for the no treatment health state (INMB range, US$40,669.30 at a multiplier of 1.25 to US$103,136.50 at a multiplier of 2.31).

Across all one-way analyses, the highest INMB was observed under the maximum utility multiplier for the early cohort (US$127,902.94), whereas the lowest INMB occurred under a 1-year time horizon (US$3579.95). INMB estimates across time horizons ranging from 1 to 25 years are shown in Supplementary Fig. S4.

### Scenario analyses

Scenario analyses supported the robustness of the base case findings, with early initiation remaining dominant or cost-effective across all scenarios including alternative discount rates and response-assessment windows (Table 1). When both strategies were assigned identical index DMARD distributions, early initiation remained dominant (INMB US$94,600.75, ICER −US$102,581.85/QALY). Biologic acquisition-cost reduction scenarios also preserved cost-effectiveness: INMB US$57,485.86 with a 15% WAC reduction and US$65,140.24 with a 60% WAC reduction. In the drug-specific net-price scenario, early initiation remained dominant (INMB US$93,738.47, ICER −US$117,390.13/QALY).

### Quantitative bias analysis

The E-value for the base case MDA relative risk (1.88) was 3.17, and 1.49 for the lower 95% confidence limit (1.12). Thus, an unmeasured confounder would need to be associated with both early initiation and MDA attainment by a risk ratio of at least 3.17 (or 1.49 at the lower confidence limit), beyond measured covariates, to fully explain away the observed association.

In the standardized attenuation analysis, all inputs were held constant except the early-versus-delayed MDA relative risk. INMB declined monotonically as the MDA relative risk was attenuated toward the null, but remained positive until the observed MDA advantage was completely removed at RR = 1.00 (Supplementary Table S11). Thus, partial attenuation of the observed MDA association did not eliminate the favourable economic result.

### Transportability analysis

The transportability stress test did not change the conclusion. When included non-U.S.-derived inputs were increased by 50%, early initiation remained dominant, with an INMB of US$57,541.36 and an ICER of −US$36,541.36/QALY. When these inputs were decreased by 50%, early initiation also remained dominant, with an INMB of US$74,786.27 and an ICER of −US$45,286.27/QALY. These findings indicate that the economic advantage of early initiation was preserved under substantial variation in non-U.S.-derived clinical, utility, mortality, and treatment-pattern inputs.

## Discussion

In this cost-utility analysis comparing early versus delayed DMARD initiation in DMARD-naive adults with PsA, early initiation was associated with greater QALYs and lower total direct medical costs over 15 years, rendering it the dominant strategy under base case assumptions. Early initiation provided an incremental gain of 0.56 QALYs at an incremental cost of –US$43,902.94 per patient, corresponding to an ICER of –US$78,398.11/QALY and a mean INMB of US$71,902.94 at a WTP of US$50,000/QALY.

The economic advantage of early DMARD initiation was driven primarily by reduced cumulative time spent in low-utility, high-cost disease states rather than by assumptions of superior efficacy for any specific drug class. By increasing the probability of earlier disease control, early initiation reduces prolonged exposure to non-response and no treatment states, which are associated with worse functional status and greater downstream healthcare utilisation.^3^^,^^4^ Transition probabilities determined the proportion of the cohort occupying each health state over time; costs and utilities were then applied to those states. Because secondary medical costs were linked to functional trajectories through HAQ-DI based cost adjustments and health benefits were captured using EQ-5D utilities, modest differences in the timing of response translated into meaningful cumulative differences in costs and QALYs. Early treatment was less costly not because of shorter treatment duration but because improved disease control reduced downstream medical costs linked to functional impairment and treatment failure.^3^^,^^11^

Several considerations support the plausibility that the observed MDA advantage reflects a true treatment-timing effect. The early-versus-delayed MDA relative risk was derived from registry data adjusted for age, sex, employment, education, smoking, body mass index, axial PsA, psoriasis, diabetes, metabolic syndrome, baseline disease status, concomitant therapy, and therapy class.^3^ The E-value of 3.17 indicates that an unmeasured confounder would need to be associated with both early initiation and MDA attainment by a risk ratio exceeding 3.17, beyond measured covariates, to fully explain away the association. This represents a high threshold relative to recognised confounders in PsA, and the standardized attenuation analysis showed that the economic conclusion reversed only when the entire observed MDA advantage was removed. The direction of effect is also biologically consistent with window-of-opportunity evidence across immune-mediated diseases. Early intensive treatment has improved outcomes in early PsA (TICOPA), Crohn’s disease (CALM), and new-onset or short-duration psoriasis (GUIDE), and similar concepts are increasingly recognised in hidradenitis suppurativa, where uncontrolled inflammation can lead to irreversible structural damage.^7^^,^^26^, ^27^, ^28^ These mechanisms, including immune memory, persistent inflammation, and tissue damage, provide biological support for a treatment-timing effect that is not solely explained by patient characteristics influencing treatment selection. The most appropriate interpretation of the findings is therefore that, if the cohort-derived MDA difference reflects a true treatment-timing effect, early initiation is economically favourable under prevailing U.S. pricing.

Class- and agent-stratified results should be interpreted as secondary analyses exploring heterogeneity in economic outcomes under a common health-state framework, not as head-to-head comparisons of clinical effectiveness. Table 1 shows early versus delayed initiation of the same DMARD class or agent. The high INMB observed for methotrexate was driven primarily by its low acquisition cost under current pricing, not by superior clinical efficacy. In that comparison, the health gain reflects earlier MDA-defined disease control, while the cost advantage reflects low methotrexate acquisition costs together with lower downstream medical costs associated with improved functional status and treatment response. Higher-cost biologic classes generated smaller INMBs or ICERs closer to conventional willingness-to-pay thresholds despite established clinical efficacy across PsA domains, underscoring the influence of acquisition costs on class-specific estimates.

Pricing scenarios further showed that drug-acquisition assumptions affected the magnitude, but not the direction, of the economic result (Table 1). Uniform 15% and 60% biologic WAC reductions lowered INMB relative to the base case because delayed initiation had greater biologic exposure in the observed treatment mix, so reducing biologic prices narrowed part of early initiation’s cost advantage. In contrast, the drug-specific net-price scenario applied different discounts by agent rather than a uniform biologic reduction. Because early and delayed strategies differed in the specific therapies used and in time accrued on treatment, these agent-specific discounts changed the weighted acquisition costs differently across strategies. In the model, this increased the incremental cost saving associated with early initiation, yielding a higher INMB than the base case. Overall, early initiation remained economically favourable across pricing scenarios, but class-specific findings should be interpreted as pricing-sensitive economic estimates rather than comparative therapeutic rankings.

Comparative clinical efficacy of therapies may also differ according to the degree of cutaneous involvement in patients with PsA, as some biologic classes demonstrate greater effectiveness in skin-dominant conditions compared to musculoskeletal manifestations alone.^29^ As such, class-specific economic findings should take into consideration underlying disease presentation and may not capture domain-specific benefits relevant to individual patients. These results underscore an important distinction for interpretation: economic dominance in a cost-utility framework reflects the balance of costs and QALYs under prevailing pricing and utilization assumptions and should not be construed as clinical superiority, particularly for patients in whom specific domains (e.g., severe skin disease, axial disease, enthesitis) drive treatment choice.

This analysis addresses a policy-relevant question that is often central to real-world access and reimbursement decisions: whether stepwise treatment pathways that prolong uncontrolled disease deliver value once long-term costs and outcomes are considered. Our findings align with emerging economic evaluations in immune-mediated diseases suggesting that earlier disease-modifying or targeted therapy can generate long-term value by improving disease control, preserving health-related quality of life, and reducing downstream costs.^30^ In PsA, reducing diagnostic delay, accelerating specialist referral, and enabling timely initiation of indicated DMARD therapy may therefore improve value by limiting time in high-burden disease states.

### Strengths and limitations

The key strength of this study was the methodological rigour of the state-transition model. The model was informed by a targeted synthesis of contemporary trial, cohort, and claims-based evidence; incorporated health utilities derived from EQ-5D; and captured downstream medical costs through functional status–linked cost adjustments using HAQ-DI. The analytic focus on DMARD-naive patients strengthens interpretability by reducing confounding from prior treatment exposure and aligns with the policy question of whether earlier initiation, rather than delayed escalation, provides better long-term value.

This study also has limitations. First, residual confounding by indication in the observational treatment-timing inputs cannot be excluded. To address this uncertainty, we conducted E-value and standardized attenuation analyses, both of which supported the robustness of the economic conclusion. The biological plausibility of a treatment-timing effect in PsA is further strengthened by converging translational, observational, and emerging trial evidence, although clinical equipoise remains regarding its precise magnitude. Accordingly, findings are best interpreted conditionally until further evaluated through disease-duration-stratified analyses of existing phase III trial data.

Second, treatment timing was categorised as ≤12 versus >12 months to match the primary source registry. Patient-level time-to-initiation data were unavailable, precluding continuous delay-response modelling, estimation of incremental costs or QALYs per additional month of delay, or subgroup-specific analyses by age, sex, comorbidity, or disease phenotype. Sensitivity to the response-assessment window was examined using 3- and 12-month alternatives, with consistent results.

Third, several clinical and utility inputs were derived from non-U.S. cohorts, although U.S. data were prioritized wherever available. To assess transportability, we varied all non-U.S.-derived inputs by ±50%. Early initiation remained economically favourable under both extremes, supporting the robustness of the findings across substantial variation in these inputs.

Fourth, base case drug acquisition costs used wholesale acquisition cost, which may overstate realized U.S. prices given confidential rebates and biosimilar competition. To address this, we modelled uniform 15% and 60% WAC reductions and a drug-specific net-price scenario applying published list-to-net discounts. Early initiation remained economically favourable across all pricing scenarios, and the net-price scenario yielded a higher INMB than the base case, suggesting that WAC-based pricing is more likely to underestimate than overestimate the cost-effectiveness of early initiation.

Fifth, individual PsA disease domains were not modelled as distinct subgroups. Their effects were captured indirectly through MDA-defined response states, EQ-5D utilities, and HAQ-DI–linked cost adjustments, although benefits in domains less well represented by these instruments may be underestimated.

Sixth, treatment sequencing and combination csDMARD therapy were simplified owing to limited long-term comparative data. However, post-switch effectiveness was informed by real-world evidence and adjusted for prior treatment exposure, and conservative shared waning parameters were applied beyond observed follow-up.

Finally, the U.S. payer perspective excludes indirect costs such as productivity loss and caregiver burden. Including these costs would plausibly strengthen the economic case for earlier disease control.

### Conclusion

In this model-based economic evaluation of early versus delayed initiation of DMARDs in DMARD-naive adults with PsA, early DMARD initiation was less costly and more effective relative to delayed initiation, making it the dominant strategy. These findings suggest that early DMARD initiation could deliver substantial health and economic value from a U.S. payer perspective. Stratified analyses of existing phase III trial data by disease duration would help confirm the magnitude of the treatment-timing effect and further strengthen the evidence base for clinical practice, guideline development, and reimbursement frameworks that currently restrict timely access to DMARD therapy.

## Contributors

SHu conceptualized and designed the study. SHu and JC performed data curation, formal analysis, interpretation, and drafted the original manuscript. EN provided supervision and oversight. SHu and EN were responsible for the decision to submit. SHu, JC, SHa, MO, MK, ER, SRG, DDG, JR, AM, AT, and EN contributed to the interpretation and critical revision of the manuscript for important intellectual content and approved the final version. SHu, JC, and EN directly accessed and verified the data. All authors had full access to the data and accept responsibility for the decision to submit.

## Data sharing statement

No individual participant data were collected or used in this study. With publication, the model input dataset, data dictionary, analytic code, and model outputs will be made available upon reasonable written request to the corresponding author. These materials will be shared with researchers whose proposed use has been approved by the study investigators for the purpose of reproducing, validating, or extending the reported cost-effectiveness analyses. Data and code will be provided by email, with investigator support as needed.

## Declaration of interests

MO received grants and/or consultation fees from AstraZeneca, Boehringer Ingelheim, and Therakos. DDG received grants and/or consultation fees from AbbVie, Amgen, BMS, Eli Lilly, Fresenius Kabi, Janssen, Johnson & Johnson, Novartis, Oruka, Pfizer and UCB. JR received grants and/or consultation fees from AbbVie, Alumis, Amgen, Apogee, Arcutis, Aristea, Aslan Therapeutics, Bausch Health, Boehringer Ingelheim, Bristol Myers Squibb, Celgene, Concert Pharma, DICE Therapeutics, Eli Lilly, Galderma, incyte, Innovaderm, JAMP Pharma, Janssen, Knight Therapeutics, Kymera Therapeutics, Kyowa Kirin, LéoPharma, L’Oréal, Merck, Novartis, Organon, Oruka Therapeutics, Pfizer, Sandoz, Sanofi, SunPharma, Takeda, and UCB. JR also serves as Treasurer of the Canadian Dermatology Association. AM received consulting fees and/or honoraria from UCB, Novartis, AbbVie, Johnson & Johnson, Bausch Health, Celltrion, and BioJamp. AT received consultation fees (honorarium) from AbbVie, Johnson & Johnson, UCB, Fresenius Kabi, and Novartis. EN received honoraria and/or consultation fees and/or participated on advisory boards for AbbVie, Apogee, Arcutis, Bausch Health, BioJamp, Boehringer Ingelheim International, Bristol Myers Squibb, Celltrion, Eli Lilly, Galderma, Innovaderm, Janssen, LEO Pharma, Medexus, Novartis Pharmaceuticals, Organon, Pfizer, Sanofi Genzyme, Searchlight Pharma, Sun Pharmaceuticals, and UCB. EN also holds unpaid leadership roles with the Canadian Dermatology Association, Global Psoriasis Atlas, and SkIN Canada.

The remaining authors have no conflicts of interest to declare.
