# Supplementary material for: Cost-effectiveness of early versus delayed disease-modifying antirheumatic drugs for psoriatic arthritis
Source: Lancet Reg Health Am. 2026 Oct 14;65:101661. doi: 10.1016/j.lana.2026.101661 (PMC13693057; doi:10.1016/j.lana.2026.101661)
Supplement: Supplementary file 1 [file mmc1.pdf]

Supplementary Appendix

Study Title: Cost-Effectiveness of Early versus Delayed Disease-Modifying Antirheumatic Drugs for Psoriatic Arthritis

Supplementary Figure 1. PRISMA Flow Diagram
Supplementary Figure 2. One-Way Sensitivity Analysis of Incremental Costs
Supplementary Figure 3. One-Way Sensitivity Analysis of Incremental QALYs
Supplementary Figure 4. Trajectory of Incremental Net Monetary Benefit Over Time

Supplementary Table 1. Glossary of Health Economic Terms
Supplementary Table 2. Search Strategy
Supplementary Table 3. PICO Framework
Supplementary Table 4. Modelled Dosing Regimens
Supplementary Table 5. Model Parameters, Distributions, and Assumptions
Supplementary Table 6. Secondary Medical Costs Composition
Supplementary Table 7. Transportability Stress Test Inputs
Supplementary Table 8. ROBINS-I Assessment
Supplementary Table 9. Risk of Bias 2 Assessment
Supplementary Table 10. Health State Occupancy Over Time
Supplementary Table 11. Standardized Attenuation Analysis of MDA RR

Supplementary Figure 1. PRISMA Flow Diagram.

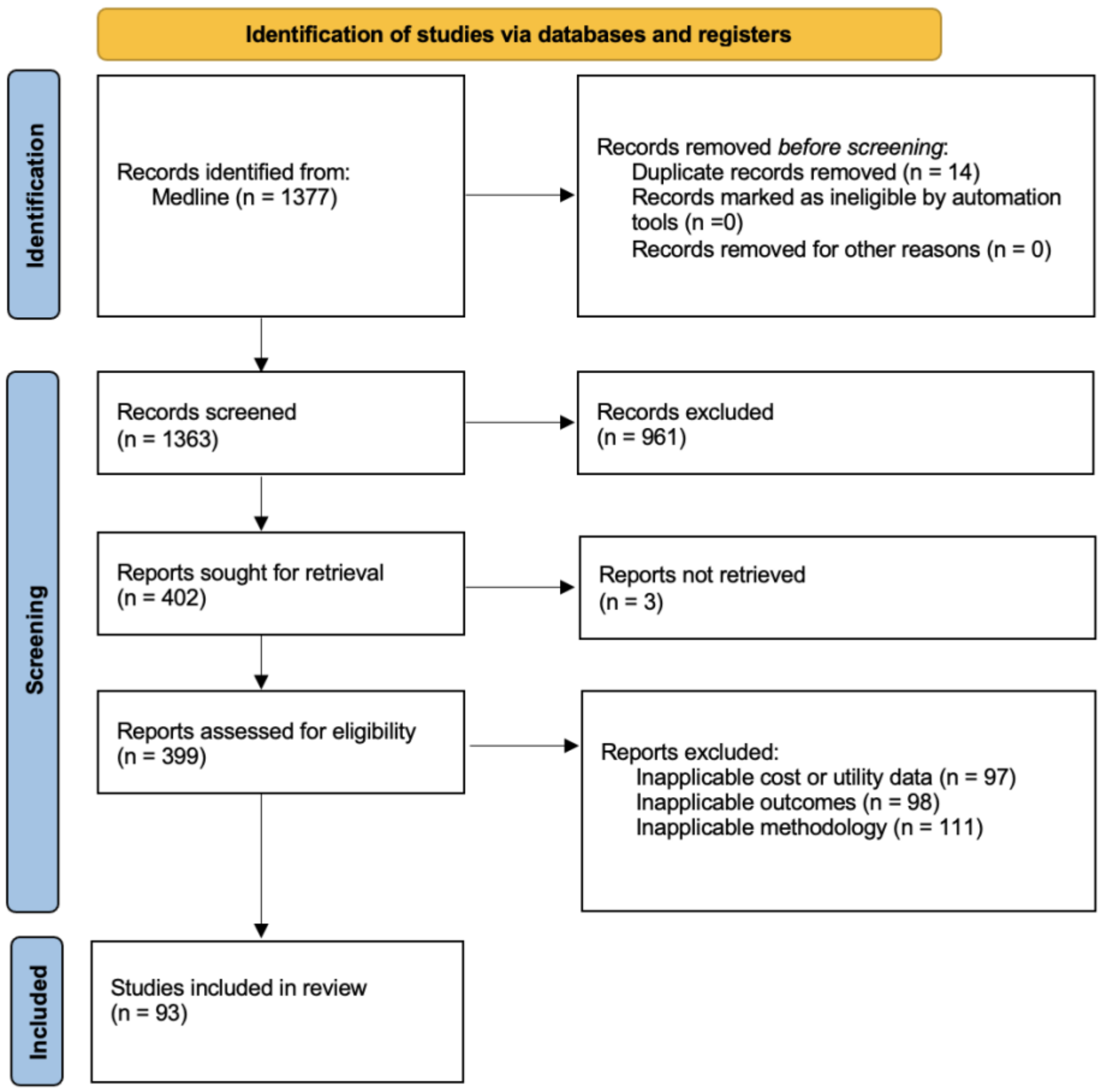

Supplementary Figure 2. One-Way Sensitivity Analysis of Incremental Costs

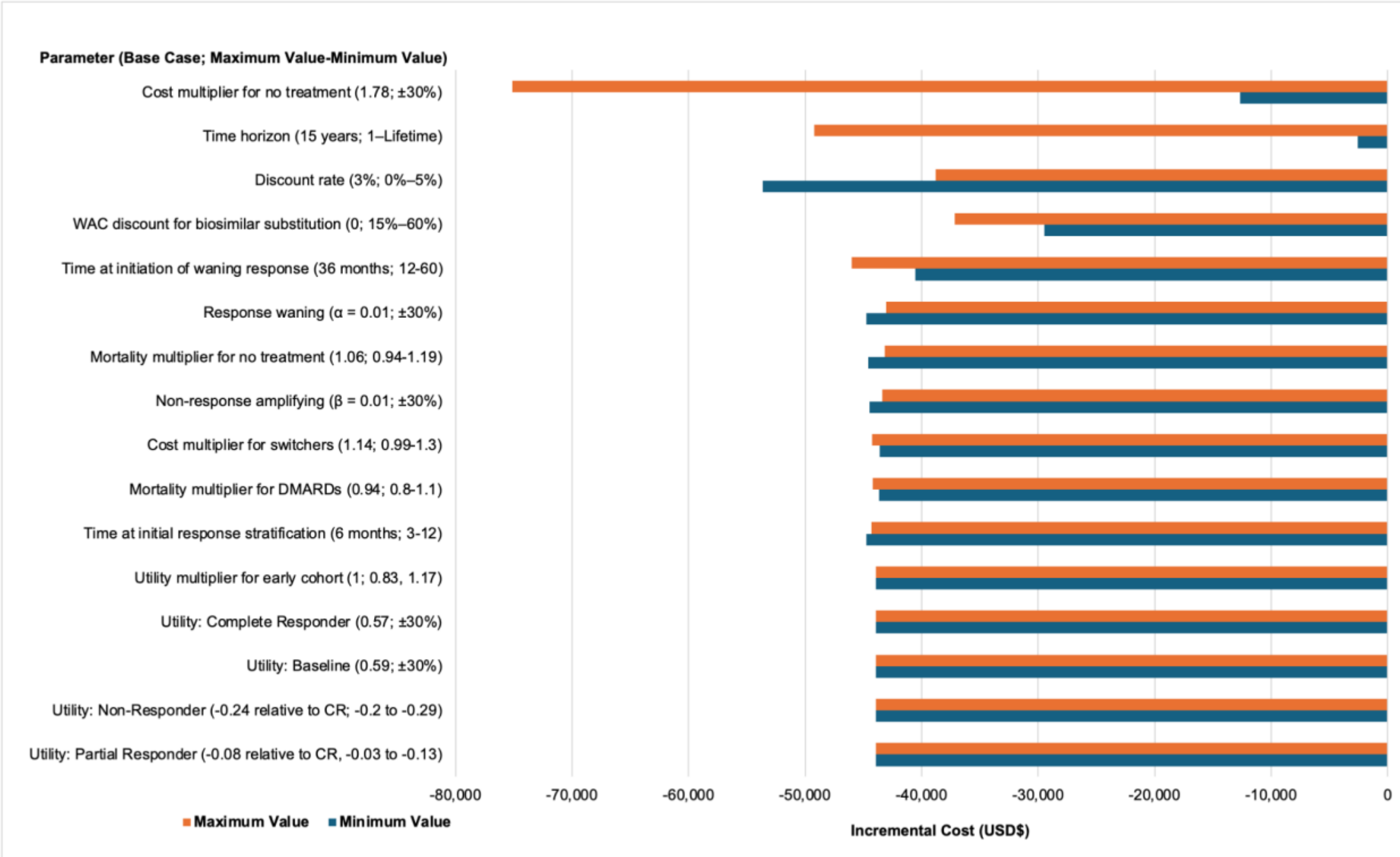

Legend. One-way sensitivity analysis showing the impact of varying key model parameters on the incremental direct medical costs of early versus delayed disease-modifying antirheumatic drug initiation. Parameters are displayed on the vertical axis with their associated minimum and maximum values, and the horizontal axis reflects incremental cost. Negative values indicate that early initiation is less costly than delayed initiation. Bars represent incremental costs when parameters are set to their minimum (blue) and maximum (orange) values.

Abbreviations: QALY, quality-adjusted life-year; US$, United States Dollar; WAC, wholesale acquisition cost.

Supplementary Figure 3. One-Way Sensitivity Analysis of Incremental QALYs

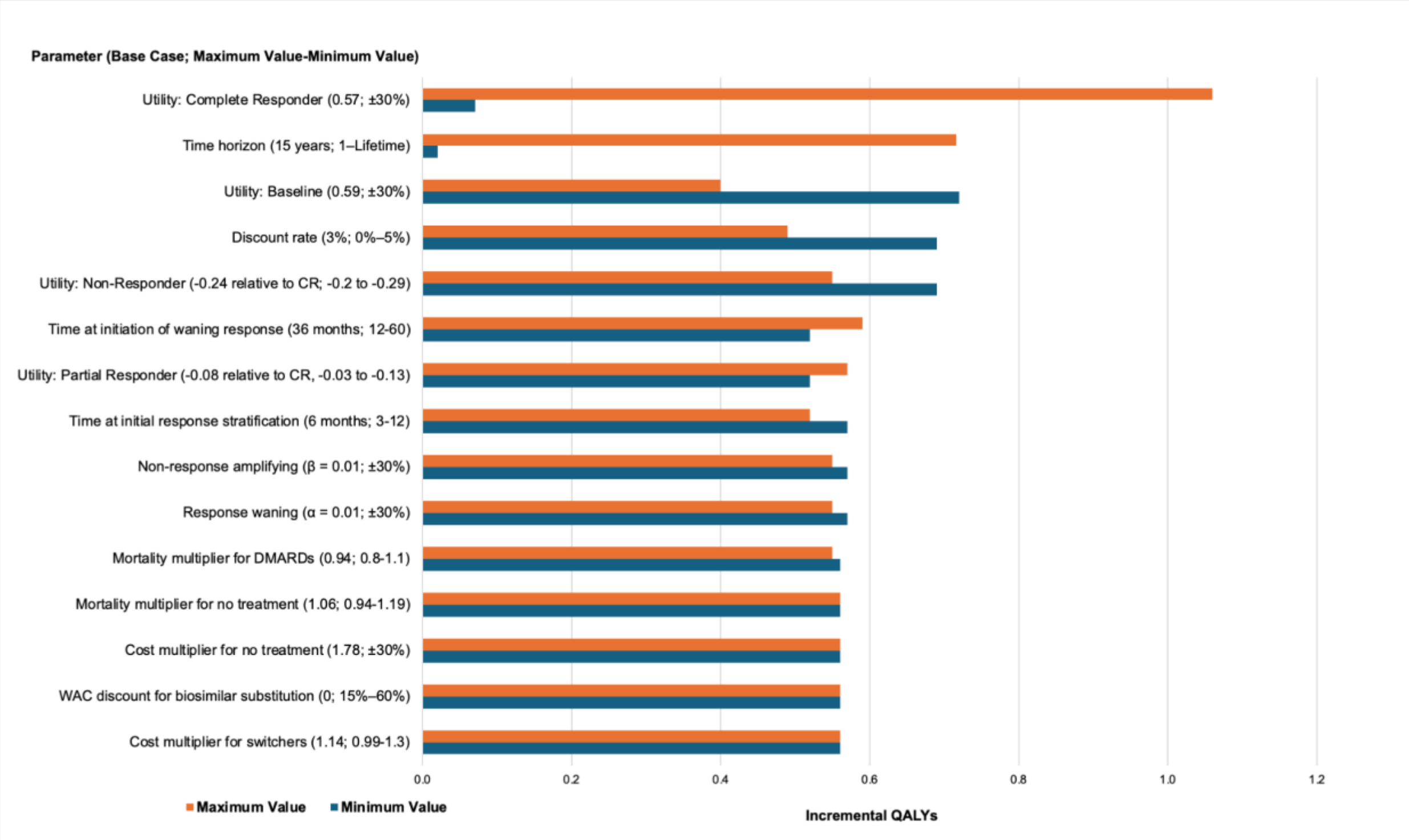

Legend. One-way sensitivity analysis showing the impact of varying key model parameters on the incremental quality-adjusted life-years (QALYs) of early versus delayed disease-modifying antirheumatic drug initiation. Parameters and their minimum and maximum values are shown on the vertical axis. The horizontal axis displays incremental QALYs. Bars represent incremental QALYs when parameters are set to their minimum (blue) and maximum (orange) values, demonstrating the parameters contributing most to uncertainty in effectiveness.

Abbreviations: QALY, quality-adjusted life-year; WAC, wholesale acquisition cost.

Supplementary Figure 4. Trajectory of Incremental Net Monetary Benefit Over Time

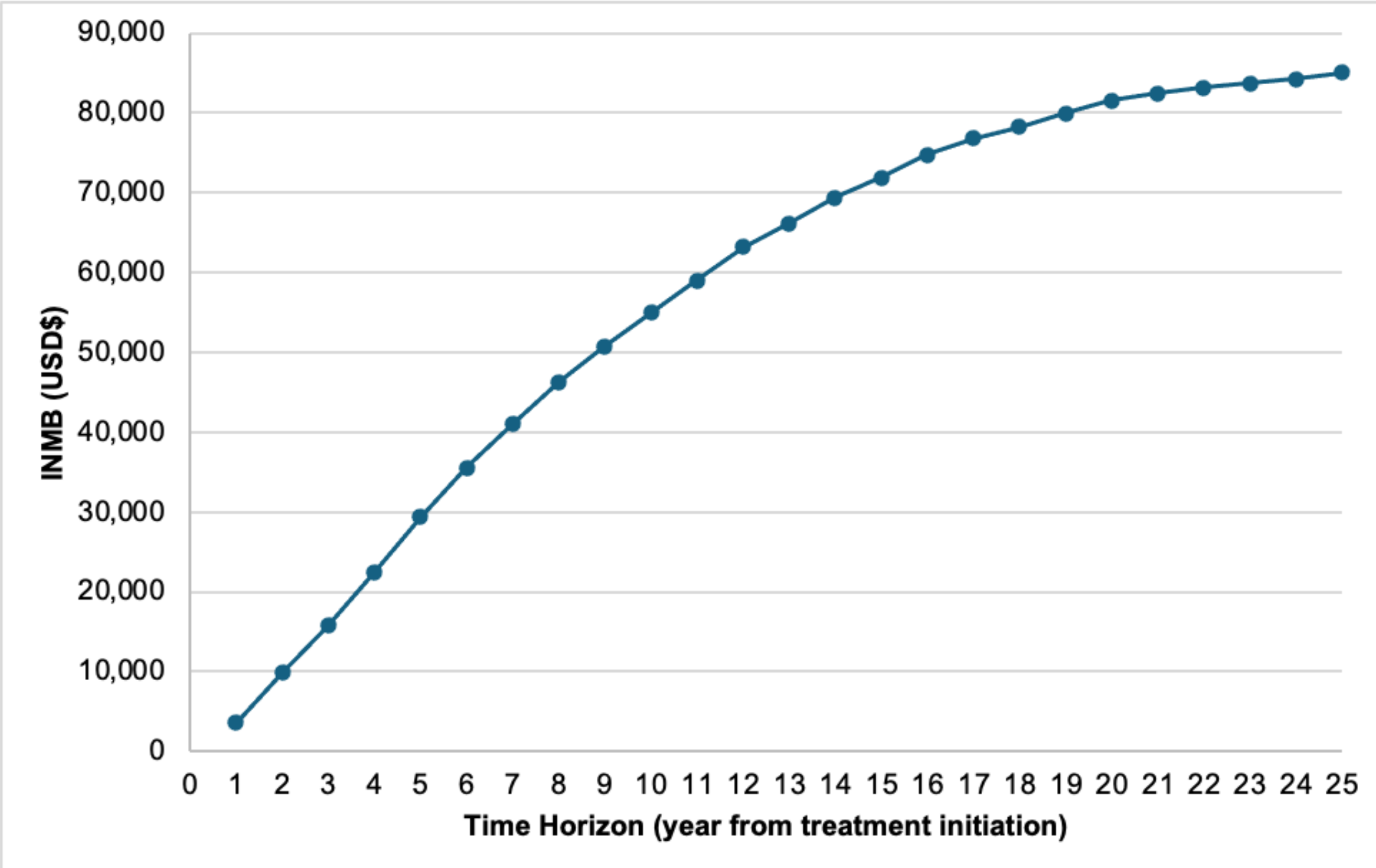

Legend. Incremental net monetary benefit (INMB) of early versus delayed disease-modifying antirheumatic drug initiation evaluated at cumulative time horizons from 1 to 25 years. The curve depicts the INMB at each horizon, reflecting how the net monetary benefit changes as costs and health outcomes accrue over longer durations.

Abbreviations: INMB, incremental net monetary benefit; US$, United States Dollar.

Supplementary Table 1. Glossary of Health Economic Terms

Cost-utility analysis (CUA)	An economic evaluation comparing the additional costs of an intervention with its additional health benefit, expressed in quality-adjusted life-years. CUAs inform whether incremental gains justify incremental costs.
Perspective	The analytic viewpoint that determines which costs and outcomes are included in the evaluation. This study used a U.S. payer perspective, which excludes indirect costs such as productivity losses.
Quality-adjusted life-year (QALY)	A measure combining the quantity and quality of life, where one QALY represents one year lived in perfect health. QALYs are used to quantify health outcomes in CUAs.
Incremental cost-effectiveness ratio (ICER)[a]	A ratio representing the additional cost per additional QALY gained by one intervention compared with another.
Dominant choice	An intervention that is more effective (e.g., yields more QALYs) and less costly than the comparator, and is therefore the preferred option.
Willingness-to-pay (WTP) threshold	The maximum amount a health system or entity is willing to spend for one additional QALY. Interventions with ICERs below the threshold are generally considered cost-effective. A threshold of USD$50,000/QALY is commonly used in the U.S.
Incremental net monetary benefit (INMB)[b]	A monetary measure of cost-effectiveness calculated by converting incremental QALYs into monetary units using a specified WTP threshold and subtracting incremental costs. A positive INMB indicates that the intervention yields net value and is cost-effective at the chosen threshold.
Transition probability	The probability of moving from one health state to another during a given cycle of a Markov model.
Health utility	A numerical value (0 to 1) reflecting an individual's preference for a specific health state, where 0 represents death and 1 represents perfect health.
Direct medical costs	Expenditures directly associated with the material or human resources required for disease management. This may include medication, hospitalization, outpatient visits, or other healthcare services.

[a]ICER = $\frac{Cost_{early} - Cost_{delayed}}{QALY_{early} - QALY_{delayed}}$

[b]INMB = (ΔQALYs*WTP) - ΔCosts

Abbreviations: CUA, cost-utility analysis; ICER, incremental cost-effectiveness ratio; INMB, incremental net monetary benefit; QALY, quality-adjusted life-year; WTP, willingness-to-pay.

Supplementary Table 2. Search Strategy

Area of Interest	Search Strategy	Hits
Intervention & Comparator Specific Data		
	("psoriatic arthritis"[MeSH Terms]) AND (("antirheumatic agents"[All Fields]) OR ("biologics"[All Fields] OR "tumor necrosis factor inhibitor"[All Fields] OR "tumor necrosis factor-alpha"[All Fields] OR "interleukin inhibitors"[All Fields] OR "interleukin-17"[All Fields] OR "interleukin-12"[All Fields] OR "interleukin-23"[All Fields] OR "janus kinase inhibitors"[All Fields])) OR ("methotrexate"[All Fields] OR "sulfasalazine"[All Fields] OR "leflunomide"[All Fields] OR "cyclosporine"[All Fields]) OR ("apremilast"[All Fields] OR "tofacitinib"[All Fields])	3935
Clinical Outcomes	AND ("flare"[All Fields] OR "recurrence"[All Fields] OR "complications"[All Fields] OR "adverse events"[All Fields] OR "side effects"[All Fields] OR "remission"[All Fields] OR “minimal disease activity”[All Fields] OR “mortality”[All Fields])	1400
Utilities	OR ("EQ-5D"[All Fields] OR "utility"[All Fields] OR "utility weight"[All Fields])	1458
Economic Models	OR ("cost-utility analysis"[All Fields] OR "cost-benefit analysis"[All Fields] OR "cost-effectiveness analysis"[All Fields] OR "economic evaluation"[All Fields])	1496
Resource Use	OR ("health resources"[All Fields] OR "hospitalization"[All Fields] OR "emergency service"[All Fields] OR "economic burden”[All Fields] OR “outpatient visit”[All Fields] OR “wholesale acquisition cost”[All Fields] OR "drug costs"[All Fields])	1513
Limitations	AND English[lang] AND (“2000/01/01”[PDAT] : “2025/12/15"[PDAT])	1377

Supplementary Table 3. PICO Framework

	**Inclusion**	**Exclusion**
Population	- Patients aged ≥ 18 years<br>- Diagnosis of PsA by a rheumatologist<br>- Patients for whom systemic therapy clinically indicated<br>- DMARD-naive at first DMARD initiation	- Pediatric populations<br>- Non-PsA inflammatory arthritis<br>- PsA treated only with NSAIDs or local therapy<br>- Pregnancy or breastfeeding
Intervention (Early)	- DMARD initiation within 12 months of PsA diagnosis	- DMARDs used for non-PsA indications or in combination with other biologics
Comparator (Delayed)	- DMARD initiation >12 months after PsA diagnosis	- DMARDs used for non-PsA indications or in combination with other biologics
Intervention & Comparator Outcomes	- Response rates<br>- Flare rates<br>- Adverse event rates<br>- Discontinuation rates<br>- Remission rates<br>- Healthcare resource utilization	- If outcomes of interest are not reported<br>- Immunologic or molecular outcomes<br>- Outcomes focused on treatments other than the intervention or comparator
PsA Outcomes	- Utility weight, EQ-5D index score<br>- Mortality rate (all-cause)<br>- Minimal disease activity<br>- Direct medical costs	- If outcomes of interest are not reported<br>- Outcomes for conditions other than PsA
Study Design	- Systematic reviews, meta-analyses<br>- RCT, cohort studies, case-control studies<br>- Economic papers or utility values involving adults with PsA<br>- English language only<br>- Studies published in the last 25 years (2000 – 2025)	- Narrative reviews, commentaries, opinions, editorials, letters without primary data<br>- Published in languages other than English<br>- Studies focused on conditions other than PsA<br>- Studies focused on PsA comorbidities<br>- Qualitative studies

Abbreviations: CASPAR, Classification Criteria for Psoriatic Arthritis; DMARD, disease-modifying antirheumatic drug; EQ-5D, EuroQol-5 Dimensions; HAQ-DI, Health Assessment Questionnaire Disability Index; ICER, incremental cost-effectiveness ratio; INMB, incremental net monetary benefit; MDA, minimal disease activity; NSAID, nonsteroidal anti-inflammatory drug; PsA, psoriatic arthritis; QALY, quality-adjusted life-year.

Supplementary Table 4. Modelled Dosing Regimens and Wholesale Acquisition Costs

Agent	DMARD Class	Unit	Per-unit WAC (US$)[1]	Modelled regimen for cost accrual
Methotrexate	csDMARD	20 mg PO	23.86	20 mg PO once weekly
Apremilast	tsDMARD	30 mg PO	88.73	30 mg PO twice daily
Tofacitinib	tsDMARD	5 mg PO	101.22	5 mg PO twice daily
Upadacitinib	tsDMARD (JAK)	15 mg PO	225.09	15 mg PO once daily
Adalimumab	bDMARD (TNFi)	40mg SC	3,461.31	40 mg SC every 2 weeks
Etanercept	bDMARD (TNFi)	50mg SC	2,039.40	50 mg SC once weekly
Certolizumab pegol	bDMARD (TNFi)	200mg SC	2,999.72	400 mg SC at weeks 0, 2, and 4, then 200 mg SC every 2 weeks
Golimumab	bDMARD (TNFi)	50mg SC	6,663.54	50 mg SC every 4 weeks
Infliximab	bDMARD (TNFi)	5 mg/kg IV	1,900.0 (assume 80kg)	5 mg/kg IV at weeks 0, 2, and 6, then every 8 weeks
Ixekizumab	bDMARD (IL-17)	80 mg SC	7,261.51	160 mg SC at week 0, 80 mg at weeks 2, 4, 6, 8, 10, and 12, then 80 mg SC every 2 weeks
Secukinumab	bDMARD (IL-17)	150 mg SC	2,507.54	150 mg SC at weeks 0, 1, 2, 3, and 4, then every 4 weeks
Ustekinumab	bDMARD (IL-12/23)	45 mg SC	6,617.67	45 mg SC at weeks 0 and 4, then every 12 weeks
Guselkumab	bDMARD (IL-23)	100 mg SC	6,936.13	100 mg SC at weeks 0 and 4, then 100 mg every 4 weeks
Bimekizumab	bDMARD (IL-17)	160 mg SC	7,922.89	160 mg SC every 4 weeks

Legend: Table summarizes the DMARD agents included in the model, their therapeutic class, unit strength and route of administration, January 2025 brand-name US wholesale acquisition cost, and modelled dosing regimen used to calculate primary index-DMARD acquisition costs. For agents with induction dosing, costs were accrued according to induction and maintenance schedules over model cycles. WAC does not reflect confidential rebates, discounts, or net prices.

Abbreviations: bDMARD, biologic disease-modifying antirheumatic drug; csDMARD, conventional synthetic disease-modifying antirheumatic drug; DMARD, disease-modifying antirheumatic drug; IL, interleukin; IV, intravenous; JAK, Janus kinase; PO, oral; SC, subcutaneous; TNFi, tumor necrosis factor inhibitor; tsDMARD, targeted synthetic disease-modifying antirheumatic drug; WAC, wholesale acquisition cost.

Supplementary Table 5. Model Parameters, Distributions, and Assumptions

Parameter	Base Value	Range Over Time	Distribution	Source	Assumptions
**Utilities**					
Baseline EQ-5D utility	0.59	Fixed	Beta	[2]	Derived from a meta-analysis of psoriatic disease cohorts in Europe and North America. This value was applied at model entry for untreated health states and held constant over time due to lack of longitudinal untreated PsA utility data. No U.S. EQ-5D estimates for untreated PsA were identified.
Utility decrement: PR vs CR	-0.08	Fixed	Normal	[3]	Estimated from a Dutch longitudinal PsA cohort reporting EQ-5D utilities stratified by durability of minimal disease activity (MDA) over 1-3 years. Sustained MDA was mapped to complete response (CR), non-sustained MDA was mapped to partial response (PR), and no MDA was mapped to non-response (NR). The adjusted mean EQ-5D difference between these groups was applied as a decrement relative to CR utility in each model cycle. No U.S. studies reporting EQ-5D utilities stratified by MDA durability were identified.
Utility decrement: NR vs CR	-0.24	Fixed	Normal		
Early cohort utility multiplier (scenario analysis)	N/A	Scenario: 1.17	Log normal	[4]	Informed by a UK-based analysis of etanercept-treated adults with PsA comparing EQ-5D change by disease duration at treatment initiation, with greater improvement observed among patients with shorter disease duration. Applied as a multiplicative adjustment to health state utilities in the early cohort to test the impact of residual health benefits not fully captured by modeled response states. Restricted to scenario analysis due to therapy-specific and non-U.S. data.
**Clinical Inputs**					
Mortality					
Standardized mortality ratio (SMR) for PsA on DMARD	0.94	Fixed	Log normal	[5]	Derived from a UK population-based cohort comparing mortality in adults with PsA receiving and not receiving DMARD therapy with matched population controls. DMARD-exposure-specific SMRs were applied to on-treatment and off-treatment health states to reflect differential mortality by treatment status.
SMR for PsA without DMARD	1.06	Fixed	Log normal		
North America SMR adjustment	1.01	Fixed	Log normal	[6]	US life-table mortality rates were scaled using North America specific PsA SMR from international PsA mortality meta-analysis to align inputs with the US payer perspective.

Initial Health-State Assignments					
Relative risk for MDA attainment: early vs delayed	1.88 (cycle 6)	1.76-1.88	Log normal	[7,8]	Derived from observational PsA cohorts reporting MDA attainment by treatment timing. The 6-month early versus delayed MDA relative risk from a US registry was applied multiplicatively to drug-specific MDA probabilities for initial response assignment at cycle 6. Longitudinal delay-stratified cohort data were used to update the relative risk through 36 months, with early treatment represented by shorter-delay strata and delayed treatment by the >1-year stratum.<br><br>MDA requires meeting at least five of the following seven criteria: tender joint count ≤1, swollen joint count ≤1, Patient Global Assessment (PtGA) of arthritis visual analogue scale (VAS) ≤20, patient pain VAS ≤15, tender entheseal point ≤ 1, body surface area ≤3% or Psoriasis Area Severity Index ≤1, and Health Assessment Questionnaire Disability Index (HAQ-DI) ≤0.5.
Relative proportion of sustained vs non-sustained MDA	1.73 (cycle 6)	1.73-1.93	Log normal	[3]	Derived from a prospective European observational PsA cohort, reporting longitudinal distribution of patients attaining sustained MDA, non-sustained MDA, and no MDA over 3 years of follow-up. This parameter was used to assign initial health states at 6 months and to inform calibration of post 6-month transition probabilities by partitioning cohort- and drug-specific MDA attainment into sustained and non-sustained response pathways.
Secondary Therapy					
Secondary DMARD initiated after treatment failure	ADA → ETA<br>CZP → SEC<br>ETA → ADA<br>GOL → SEC<br>INF → APR<br>SEC → IXE<br>UST → SEC<br>APR → ADA<br>TOF → ADA	Fixed	N/A	[9]	Second-line DMARD assignments were informed by a Swedish registry study reporting common treatment switches among adults with PsA. Agent-level switch pathways were mapped to the model according to index DMARD. Where an index agent was not reported, adalimumab was used as the default second-line therapy to maintain a consistent biologic comparator. Comparable U.S. or PsA-specific data reporting agent-level switching pathways by index DMARD were not identified.
MDA adjustment for b/tsDMARD exposure	0.69	Fixed	Log normal	[10]	Derived from a U.S. registry cohort reporting MDA among DMARD-experienced patients initiating DMARD. Adjustment was applied multiplicatively to MDA probabilities for switchers with prior b/tsDMARD exposure, reflecting lower expected response after prior advanced therapy.

MDA adjustment for prior csDMARD exposure	1.14	Fixed	Log normal	[10]	Derived from a U.S. registry cohort reporting MDA attainment among DMARD-experienced patients initiating DMARD therapy. The adjustment was applied multiplicatively to MDA probabilities for switchers with prior csDMARD exposure.
**Drug-Specific Clinical and Cost Inputs**					
Targeted Synthetic DMARDs					
Apremilast (APR) MDA response probability	35.7% (cycle 6)	35.70-48.6%	Beta	[11]	MDA rates were derived from a German observational cohort of adults with PsA initiating apremilast with standard oral induction titration then 30 mg orally twice daily maintenance, with most DMARD-naive at initiation. No comparable U.S. MDA data for apremilast were identified.
APR NR → R transition (early)	8.91%	Fixed	Beta		Transitions between non-response and response states for apremilast were informed by the same German longitudinal cohort, which reported repeated MDA assessments over follow-up of 13 months among patients treated with apremilast using the labeled induction titration followed by 30 mg orally twice daily maintenance, enabling estimation of changes in response status over time.
APR R → NR transition (early)	5.73%	Fixed	Beta		
APR NR → R transition (delayed)	4.03%	Fixed	Beta		
APR R → NR transition (delayed)	7.41%	Fixed	Beta		
Tofacitinib (TOF) MDA response probability	45.52% (cycle 6)	45.52-53%	Beta	[12]	Derived from longitudinal cohorts with North American representation enrolling adults with PsA initiating tofacitinib 5 mg orally twice daily.
TOF NR → R transition (early)	11.45%	Fixed	Beta		Transitions between non response and response states were informed by the same longitudinal cohorts reporting initial MDA outcomes, which included repeated MDA assessments over 30 months of follow-up among patients receiving tofacitinib 5 mg twice daily, allowing characterization of response durability.
TOF R → NR transition(early)	6.95%	Fixed	Beta		
TOF NR to R rate (delayed)	3.86%	Fixed	Beta		
TOF R → NR transition (delayed)	7.0%	Fixed	Beta		

Monthly switch probability	2.18% (cycle 6)	0.22-2.74%	Beta	13	Derived from a Nordic longitudinal cohort of adults with PsA receiving DMARD therapy, in which treatment transitions were reported across all DMARD classes. These rates were applied consistently across all DMARD classes in the model to define post-treatment pathways for non-response and discontinuers. Comparable long-term U.S. data reporting class-specific patterns were not identified.
Monthly discontinuation probability	7.61% (cycle 6)	1.69-9.63%	Beta		
Monthly treatment restart probability	6.74% (cycle 6)	0.64-6.74%	Beta		
Conventional Synthetic DMARD					
Methotrexate (MTX) MDA probability	29.39% (cycle 6)	29.39-36.57%	Beta	14	Derived from an international, multicenter, randomized, double-blind phase 3 trial that included a methotrexate monotherapy arm (target 20 mg per week).
MTX NR → R transition (early)	1.97%	Fixed	Beta		Monthly transitions between non-response and response were parameterized using longitudinal MDA data from the same randomized trial, with MDA outcomes reported over 12 months. These data supported estimation of post 6-month transitions between response states. Comparable U.S.-specific estimates for methotrexate monotherapy in PsA were not identified.
MTX R → NR transition (early)	0.50%	Fixed	Beta		
MTX NR → R transition (delayed)	0.94%	Fixed	Beta		
MTX R → NR transition (delayed)	0.50%	Fixed	Beta		
Monthly switch probability	0.71% (cycle 6)	0.15-0.94%	Beta	13	Monthly probabilities of switching, stopping, and restarting systemic therapy were derived from a Nordic registry based longitudinal cohort reporting treatment patterns over 120 months. Comparable long-term U.S. registry data reporting switching, discontinuation, and restart patterns in PsA were not identified.
Monthly discontinue probability	3.67% (cycle 6)	1.20-5.53%	Beta		
Monthly restart probability	7.76% (cycle 6)	0.85-3.42%	Beta		
Biologic DMARDs					
Ixekizumab (IXE) MDA response probability	25.60% (cycle 6)	25.60-39.26%	Beta	15	Derived from a multicenter, multinational, phase 3 randomized trial enrolling adults with active PsA, treated with ixekizumab 80 mg SC every 2 weeks at maintenance.
IXE NR → R transition (early)	6.44%	Fixed	Beta		Transitions were informed by the same phase 3 trial, which reported repeated MDA assessments among patients receiving

IXE R → NR transition (early)	6.80%	Fixed	Beta		ixekizumab 80 mg every 2 weeks. Transitions were thus derived from multinational trial follow-up, as no U.S.-exclusive estimates for this regimen were identified.
IXE NR → R transition (delayed)	0.19%	Fixed	Beta		
IXE R → NR transition (delayed)	0.42%	Fixed	Beta		
Etanercept (ETA) MDA response probability	39.81% (cycle 6)	39.81-51.85%	Beta	14	Derived from a multicenter, multinational, phase 3 randomized trial enrolling adults with active PsA treated with etanercept 50 mg SC once weekly as monotherapy.
ETA NR → R transition (early)	2.96%	Fixed	Beta		Transitions between non-response and response states were informed by the same phase 3 trial, which reported MDA over 12 months of follow-up among patients receiving etanercept 50 mg SC once weekly, allowing characterization of response durability beyond the initial assessment. No U.S.-exclusive estimates for this regimen were identified.
ETA R → NR transition (early)	0.50%	Fixed	Beta		
ETA NR → R transition (delayed)	1.20%	Fixed	Beta		
ETA R → NR transition (delayed)	0.50%	Fixed	Beta		
Adalimumab (ADA) MDA response probability	40.3% (cycle 6)	40.3-61%	Beta	16	Derived from a randomized, double-blind, placebo-controlled phase 3 trial enrolling adults with active PsA treated with adalimumab 40 mg SC every 2 weeks, reporting MDA through 36 months of follow-up.
ADA NR → R transition (early)	4.63%	Fixed	Beta		Post-6-month movement between response and non-response states was informed by longitudinal MDA trajectories from the same trial population, using observed durability of response to derive transition probabilities. No U.S. exclusive longitudinal MDA transition estimates were identified. Therefore, transitions were derived from multinational trial follow-up.
ADA R → NR transition (early)	0.50%	Fixed	Beta		
ADA NR → R transition (delayed)	1.71%	Fixed	Beta		
ADA R → NR transition (delayed)	0.50%	Fixed	Beta		

Certolizumab pegol (CZP) MDA response probability	37.6% (cycle 6)	37.6-59%	Beta	17	Derived from a multinational, phase 3, randomized, double-blind, placebo-controlled trial conducted across North America and Europe in adults with active PsA, including biologic-naive patients. Efficacy reported over up to 4 years for CZP 200 mg SC every 2 weeks following induction.
CZP NR → R transition (early)	1.97%	Fixed	Beta		Transitions between non-response and response states beyond the initial 6-month distribution were parameterized using longitudinal MDA durability data from the same multinational trial program. In the absence of U.S. registry–based transition data for CZP, multinational trial follow-up informed transition estimates.
CZP R → NR transition (early)	0.50%	Fixed	Beta		
CZP NR → R transition (delayed)	0.88%	Fixed	Beta		
CZP R → NR transition (delayed)	0.50%	Fixed	Beta		
Golimumab (GOL) MDA response probability	27.9% (cycle 6)	27.9-51.4%	Beta	18	Derived from a multicenter, multinational phase 3 randomized, double-blind, placebo-controlled trial enrolling adults with active PsA, including biologic-naive patients.
GOL NR → R transition (early)	2.45%	Fixed	Beta		Transitions between non-response and response states were informed by the same long-term phase 3 trial, which reported longitudinal MDA status over 64 months of follow-up. No U.S.-exclusive efficacy estimates for golimumab MDA were identified.
GOL R → NR transition (early)	0.50%	Fixed	Beta		
GOL NR → R transition (delayed)	1.13%	Fixed	Beta		
GOL R → NR transition (delayed)	0.50%	Fixed	Beta		
Guselkumab (GUS) MDA response probability	19% (cycle 6)	19-38%	Beta	19	Derived from a multicenter, multinational phase 3 randomized trial conducted in biologic-naive adults with active PsA treated with guselkumab 100 mg SC.
GUS NR → R transition (early)	2.10%	Fixed	Beta		Transitions between non-response and response states were informed by the same phase 3 trial reporting longitudinal MDA outcomes over 24 months of follow-up. No U.S.
GUS R → NR	0.50%	Fixed	Beta		

transition (early)					exclusive MDA estimates for guselkumab were identified.
GUS NR → R transition (delayed)	1.03%	Fixed	Beta		
GUS R → NR transition (delayed)	0.50%	Fixed	Beta		
Secukinumab (SEC) MDA response probability	34% (cycle 6)	34-42%	Beta	20	Derived from a multicenter, multinational, phase 3 randomized, double-blind, placebo-controlled trial enrolling adults with active PsA treated with secukinumab 150 mg SC with maintenance 150 mg SC every 4 weeks.
SEC NR → R transition (early)	1.17%	Fixed	Beta		Transitions between non-response and response states were informed by the same long-term phase 3 trial, which reported longitudinal MDA status over follow-up and was used to characterize response durability beyond the initial 6 month distribution. No U.S.-exclusive MDA estimates for this regimen were identified.
SEC R → NR transition (early)	0.50%	Fixed	Beta		
SEC NR → R transition (delayed)	0.59%	Fixed	Beta		
SEC R → NR transition (delayed)	0.50%	Fixed	Beta		
Infliximab (INF) MDA response probability	52% (cycle 6)	30-52%	Beta	21	Derived from multicenter, multinational, phase 3 randomized trial program enrolling adults with active PsA, treated with infliximab 5 mg/kg IV (induction then every 8 weeks).
INF NR → R transition (early)	0.50%	Fixed	Beta		Transitions between non-response and response states were informed by the same phase 3 randomized trial program, which reported longitudinal MDA over up to 24 months of follow-up. No U.S.-exclusive infliximab MDA estimates were identified.
INF R → NR transition (early)	1.95%	Fixed	Beta		
INF NR → R transition (delayed)	0.50%	Fixed	Beta		
INF R → NR transition (delayed)	2.25%	Fixed	Beta		

Bimekizumab (BIM) MDA response probability	81.1% (cycle 6)	61.2-81.1%	Beta	[22]	Derived from a multicenter, multinational, phase 3 randomized, double-blind, placebo-controlled trial conducted in adults with active PsA who were biologic-naïve at baseline, receiving bimekizumab 160 mg SC every 4 weeks.
BIM NR → R transition (early)	0.50%	Fixed	Beta		Transitions between non-response and response states were informed by longitudinal MDA trajectories reported within the same phase 3 trial population. No U.S. exclusive longitudinal MDA transition estimates for bimekizumab were identified.
BIM R → NR transition (early)	2.03%	Fixed	Beta		
BIM NR → R transition (delayed)	0.50%	Fixed	Beta		
BIM R → NR transition (delayed)	2.09%	Fixed	Beta		
Ustekinumab (UST) MDA response probability	28.5% (cycle 6)	28.5-41%	Beta	[23]	Derived from a European, multicenter, prospective study of PsA adults receiving ustekinumab 45 mg SC every 12 weeks, following induction at weeks 0 and 4.
UST NR → R transition (early)	2.12%	Fixed	Beta		Transitions between non-response and response states were informed by the same European real-world cohort, which reported longitudinal MDA status over 3 years of follow-up. U.S. specific longitudinal MDA estimates for ustekinumab were not available.
UST R → NR transition (early)	0.50%	Fixed	Beta		
UST NR → R transition (delayed)	0.99%	Fixed	Beta		
UST R → NR transition (delayed)	0.50%	Fixed	Beta		
Upadacitinib (UPA) MDA response probability	39.1% (cycle 6)	39.1-59%	Beta	[24]	Derived from a multicenter cohort study conducted across Germany and Switzerland, enrolling adults with active PsA treated with upadacitinib 15 mg orally once daily.
UPA NR → R transition (early)	0.45%	Fixed	Beta		Transitions were informed by the same cohort, which reported MDA over 64 months, enabling characterization of response durability beyond the initial distribution. U.S. specific MDA estimates for upadacitinib were not available.
UPA R → NR transition (early)	0.13%	Fixed	Beta		

UPA NR → R transition (delayed)	0.50%	Fixed	Beta		
UPA R → NR transition (delayed)	0.62%	Fixed	Beta		
Monthly switch probability	0.20% (cycle 6)	0.02-0.27%	Beta	[13]	Derived from a Nordic registry-based longitudinal cohort of adults with PsA receiving bDMARD therapy. Reported event rates were converted to monthly probabilities and applied uniformly across all biologic DMARDs in the model.
Monthly discontinue probability	2.71% (cycle 6)	0.82 -2.88%	Beta		
Monthly treatment restart probability	6.98% (cycle 6)	0.89-6.98%	Beta		

Abbreviations: ADA, adalimumab; APR, apremilast; CR, complete response; CZP, certolizumab pegol; DMARD, disease-modifying antirheumatic drug; EQ-5D, EuroQol 5-Dimensions; ETA, etanercept; GOL, golimumab; GUS, guselkumab; INF, infliximab; IXE, ixekizumab; MDA, minimal disease activity; NR, non-response; PR, partial response; PsA, psoriatic arthritis; SEC, secukinumab; SMR, standardized mortality ratio; TOF, tofacitinib; UPA, upadacitinib; US, United States; UST, ustekinumab.

Supplementary Table 6. Secondary Medical Cost Composition

Input	Calculation	Model Application	Base Case Values	Source
Primary DMARD acquisition cost	January 2025 U.S. WAC for each agent × units dispensed per cycle, per the modelled regimen.	Applied each cycle only while a patient remained on the corresponding DMARD; modelled separately from secondary healthcare costs.	Supplementary Table 4	[1]
Baseline secondary cost	Claims-derived monthly healthcare cost assigned by index DMARD or DMARD class, excluding the primary index-DMARD acquisition cost modelled separately above.	Base secondary cost applied according to DMARD. Captures downstream healthcare use including inpatient, outpatient, emergency, physician, other outpatient services, and non-index pharmacologic costs.	Mean monthly costs:<br>tsDMARD: $927.09-$1086.93<br>csDMARD: $850.31-$938.28<br>bDMARD: $3262.72-$3825.26	[25]
Time adjustment	Baseline secondary healthcare cost × year-specific medical-cost trend factor for post-index years 1-5; factor held constant thereafter.	Applied in each cycle to both early and delayed cohorts to reflect longitudinal changes in healthcare costs over follow-up from the time of DMARD initiation.	Time multiplier range: 0.90-1.17	[26]
Treatment pathway adjustment	Time-adjusted secondary healthcare cost × pathway multiplier	Applied according to treatment pathway; discontinuer multiplier applied in the no treatment state and switcher multiplier applied during switching pathways.	Pathway multipliers:<br>1.78 for discontinuers<br>1.14 for switchers<br>1.00 otherwise	[27]
HAQ-DI adjustment	Marginal medical cost of worse functional status across response states, applied as the between-state difference relative to the complete response reference state.	Added each cycle by cohort and health state; captures only the functional-status cost gradient (CR < PR < NR).	HAQ-DI medical-cost relationship:<br>×1.38 per 1-unit HAQ-DI increase<br><br>HAQ-DI cohort multiplier range:<br>0.79-1.04	[3,7,28]
Total per cycle cost	Total cost = primary DMARD acquisition cost + [(baseline secondary healthcare cost × time adjustment × pathway multiplier) + cycle-specific HAQ-DI-linked cost adjustment].		Calculated for each cohort, health state, and cycle. Primary acquisition costs and secondary healthcare costs modelled as additive components.	

Legend: This table summarizes how secondary healthcare costs were assembled in each model cycle. Secondary costs were derived from claims-based healthcare cost estimates and adjusted for time, treatment pathway, and functional status. Primary index-DMARD acquisition costs were calculated separately using WAC-based dosing inputs and then added to secondary healthcare costs to estimate total per-cycle costs.

Abbreviations: DMARD, disease-modifying antirheumatic drug; HAQ-DI, Health Assessment Questionnaire Disability Index; US, United States; WAC, wholesale acquisition cost.

Supplementary Table 7. Transportability Stress Test Inputs.

Domain	Variable	Base Inputs Varied +/- 50%	Source
Utilities	Utility decrement: PR vs CR	-0.08	3
	Utility decrement: NR vs CR	-0.24	3
	Baseline EQ-5D utility	0.59	2
Cost	HAQ-DI relative ratio, early vs delayed	0.79-1.04	7
	HAQ-DI by health state	CR 0.27 / PR 0.49 / NR 1.13 (6 month)	3
Mortality	SMR for PsA on DMARD therapy	0.94	5
	SMR for PsA not on DMARD therapy	1.06	5
Transition probabilities	Relative proportion sustained vs nonsustained MDA	1.73-1.93	3
	Apremilast - MDA response and durability	35.70-48.6%	11
	Upadacitinib - MDA response and durability	39.1-59.0%	24
	Ustekinumab - MDA response and durability	28.5-41.0%	23
	Monthly switch probability – tsDMARD	0.22-2.74%	13
	Monthly discontinuation probability – tsDMARD	1.69-9.63%	13
	Monthly restart probability – tsDMARD	0.64-6.74%	13
	Monthly switch probability – csDMARD	0.15-0.94%	13
	Monthly discontinuation probability – csDMARD	1.20-5.53%	13
	Monthly restart probability – csDMARD	0.85-3.42%	13
	Monthly switch probability – bDMARD	0.02-0.27%	13
	Monthly discontinuation probability – bDMARD	0.82 -2.88%	13
	Monthly restart probability – bDMARD	0.89-6.98%	13

Legend: Summarizes non-U.S. derived model inputs varied in the transportability stress test. Listed inputs were varied by ±50% while U.S.-derived inputs were held constant. Multinational registration trials that enrolled North American sites were treated as U.S.-applicable and were not varied.

Abbreviations: bDMARD, biologic disease-modifying antirheumatic drug; csDMARD, conventional synthetic disease-modifying antirheumatic drug; DMARD, disease-modifying antirheumatic drug; EQ-5D, EuroQol-5 Dimensions; HAQ-DI, Health Assessment Questionnaire Disability Index; MDA, minimal disease activity; PsA, psoriatic arthritis; tsDMARD, targeted synthetic disease-modifying antirheumatic drug; WAC, wholesale acquisition cost.

Supplementary Table 8. ROBINS-I Assessment

Study (Author/Year)	D1	D2	D3	D4	D5	D6	D7	Overall
Mease, 2025	Moderate	Low	Low	Moderate	Low	Low	Low	Low
Yang, 2022	Low	Low	Low	Low	Moderate	Low	Low	Low
Snoeck Henkemans, 2022	Moderate	Low	Low	Moderate	Low	Low	Low	Low
Merola, 2021	Moderate	Low	Low	Moderate	Low	Low	low	Low
Hur, 2021	Moderate	Moderate	Low	Moderate	Moderate	Low	Low	Moderate
Maksabedian Hernandez, 2020	Moderate	Moderate	Low	Moderate	Moderate	Low	Low	Moderate
Snoeck Henkemans, 2024	Moderate	Moderate	Low	Moderate	Moderate	Low	Low	Moderate
Ogdie, 2022	Moderate	Moderate	Low	Low	Moderate	Low	Low	Low
Ogdie, 2014	Moderate	Low	Low	Low	Low	Low	Low	Low
Huang, 2025	Moderate	Moderate	Low	Low	Moderate	Low	Low	Moderate
Murage, 2022	Moderate	Moderate	Low	Moderate	Moderate	Low	Low	Moderate
Ogdie, 2024	Moderate	Moderate	Low	Moderate	Moderate	Low	Low	Moderate
Behrens, 2025	Moderate	Moderate	Low	Moderate	Moderate	Low	Low	Moderate
Krantz, 2023	Moderate	Moderate	Low	Moderate	Moderate	Low	Low	Moderate
Mease, 2017	Moderate	Moderate	Low	Low	Moderate	Low	Low	Moderate
Werner, 2023	Moderate	Moderate	Low	Moderate	Moderate	Low	Low	Moderate

Legend. D1: bias due to confounding, D2: bias due to selection of participants, D3: bias in classification of interventions, D4: bias due to deviations from intended interventions, D5: bias due to missing data, D6: bias in measurement of outcomes, D7: bias in selection of the reported result.

Supplementary Table 9. Risk of Bias 2 Assessment

**Study (Author/Year)**	**D1**	**D2**	**D3**	**D4**	**D5**	**Overall**
Kirkham, 2015	Some concerns	Some concerns	Low	Low	Some concerns	Some concerns
Mease, 2019	Low	Some concerns	Low	Low	Some concerns	Some concerns
Coates, 2022	Low	Low	Low	Low	Low	Low
van der Heijde D, 2018	Low	Some concerns	Some concerns	Low	Low	Some concerns
Kavanaugh, 2016	Low	Some concerns	Some concerns	Low	Low	Some concerns
McInnes, 2022	Low	Low	Low	low	Low	Low
Coates, 2018	Low	Low	Low	Low	Low	Low
Coates, 2010	Low	Low	Low	Low	Low	Low
McInnes, 2023	Low	Low	Some concerns	Low	Low	Low
Gossec, 2023	Low	Low	Low	Low	Low	Low

Legend. D1: Bias arising from the randomization process, D2: Bias due to deviations from intended interventions, D3: Bias due to missing outcome data, D4: Bias in measurement of the outcome, D5: Bias in selection of the reported result.

Supplementary Table 10. Health state occupancy over time.

Cycle (month)	Cohort	Pre-treatment	Complete-response state	Partial-response state	Non-response state	No treatment	Death
0	Early	100%	0.0%	0.0%	0.0%	0.0%	0.0%
0	Delayed	100%	0.0%	0.0%	0.0%	0.0%	0.0%
7 (post-assignment)	Early	0.0%	28.07%	16.20%	55.68%	0.0%	0.04%
7 (post-assignment)	Delayed	0.0%	14.64%	8.44%	76.88%	0.0%	0.04%
12	Early	0.35%	28.89%	21.60%	41.18%	7.73%	0.26%
12	Delayed	0.46%	14.10%	12.58%	62.41%	10.19%	0.26%
36	Early	0.39%	36.51%	25.60%	20.88%	15.24%	1.38%
36	Delayed	0.60%	16.29%	19.86%	38.48%	23.33%	1.44%
60	Early	0.24%	38.38%	25.19%	17.38%	16.10%	2.70%
60	Delayed	0.39%	17.68%	21.43%	31.85%	25.82%	2.83%
120	Early	0.16%	29.44%	21.89%	21.68%	19.83%	7.01%
120	Delayed	0.24%	14.72%	18.92%	30.44%	28.35%	7.34%
180	Early	0.18%	17.09%	18.94%	26.78%	24.07%	12.94%
180	Delayed	0.25%	7.67%	13.88%	34.58%	30.12%	13.51%

Legend. Proportion of cohort (%) in each state at selected cycles. Values include first- plus second-line (post-switch) response sub-states.

Supplementary Table 11. Standardized Attenuation Analysis of MDA Relative Risk

MDA RR	INMB ($) at WTP US$50,000/QALY	ICER (US$/QALY)
1.88	37,550.20	−128,810.85
1.70	32,459.58	−$120,839.89
1.50	24,752.63	−$126,804.50
1.30	16,120.24	−$129,113.78
1.20	11,120.26	−$135,337.67
1.10	6,266.84	−$106,671.00
1.05	3,166.25	−$108,312.50
1.00	0	N/A

Legend. Standardized attenuation analysis in which initial response state probabilities were recalculated as the early versus delayed MDA relative risk was attenuated from 1.88 to 1.00, with all other inputs within the standardized model held fixed. INMB is reported at a willingness-to-pay threshold of US$50,000/QALY; relative risk = 1.00 represents complete attenuation of the observed timing effect.

Abbreviations: ICER, incremental cost-effectiveness ratio; INMB, incremental net monetary benefit; QALY, quality-adjusted life-year; RR, relative risk; US$, United States Dollar; WTP, willingness-to-pay.

References

1 HCPCS Drug Pricing Search Tool. https://pricing.buyandbill.com/ (accessed Dec 21, 2025).

2 Yang Z, Li S, Wang X, Chen G. Health state utility values derived from EQ-5D in psoriatic patients: a systematic review and meta-analysis. *J Dermatolog Treat* 2022; **33**: 1029–36.

3 Snoeck Henkemans SVJ, de Jong PHP, Luime JJ, *et al.* Importance of quick attainment of minimal disease activity for a positive impact on lives of patients with psoriatic arthritis. *RMD Open* 2022; **8**: e002706.

4 Kirkham B, de Vlam K, Li W, *et al.* Early treatment of psoriatic arthritis is associated with improved patient-reported outcomes: findings from the etanercept PRESTA trial. *Clin Exp Rheumatol* 2015; **33**: 11–9.

5 Ogdie A, Haynes K, Troxel AB, *et al.* Risk of mortality in patients with psoriatic arthritis, rheumatoid arthritis and psoriasis: a longitudinal cohort study. *Ann Rheum Dis* 2014; **73**: 149–53.

6 Huang H, Xie W, Geng Y, Fan Y, Zhang Z. Mortality in patients with psoriatic arthritis: a systematic review and meta-analysis. *Front Immunol* 2025; **16**: 1622159.

7 Snoeck Henkemans SVJ, de Jong PHP, Luime JJ, *et al.* Window of opportunity in psoriatic arthritis: the earlier the better? *RMD Open* 2024; **10**: e004062.

8 Mease PJ, Nowak M, Choi J, *et al.* Impact of delay of Treatment With Disease-Modifying Antirheumatic Drugs in Psoriatic Arthritis: The CorEvitas Psoriatic Arthritis/Spondyloarthritis Registry. *ACR Open Rheumatol* 2025; **7**: e70019.

9 Murage MJ, Princic N, Park J, *et al.* Treatment patterns and health care costs among patients with psoriatic arthritis treated with biologic or targeted synthetic disease-modifying antirheumatic drugs. *J Manag Care Spec Pharm* 2022; **28**: 206–17.

10 Ogdie A, Song C, Middaugh N, *et al.* Achievement of Minimal and Very Low Disease Activity Among Patients with Psoriatic Arthritis Initiating Biologic or Targeted Synthetic Disease-Modifying Antirheumatic Drugs in the CorEvitas PsA/SpA Registry. .

11 Behrens F, Wollenhaupt J, Fiene M, *et al.* Real-world effectiveness and tolerability of apremilast in psoriatic arthritis in Germany: Results from LAPIS-PsA. *Open Rheumatol J* 2025; **19**. DOI:10.2174/0118743129352940250328055132.

12 Nash P, Coates LC, Kivitz AJ, *et al.* Safety and efficacy of tofacitinib in patients with active psoriatic arthritis: Interim analysis of OPAL Balance, an open-label, long-term extension study. *Rheumatol Ther* 2020; **7**: 553–80.

13 Krantz Å, Carrero JJ, Yang Y, Schalin L, Lysell J, Mazhar F. Psoriasis/psoriatic arthritis patients' long-term treatment patterns and adherence to systemic treatments monitoring recommendations. *Acta Derm Venereol* 2023; **103**: adv6505.

14 Mease PJ, Gladman DD, Collier DH, *et al.* Etanercept and methotrexate as monotherapy or in combination for psoriatic arthritis: Primary results from a randomized, controlled phase III trial. *Arthritis Rheumatol* 2019; **71**: 1112–24.

15 Coates LC, Mease P, Kronbergs A, *et al.* Efficacy and safety of ixekizumab in patients with active psoriatic arthritis with and without concomitant conventional disease-modifying antirheumatic drugs: SPIRIT-P1 and SPIRIT-P2 3-year results. *Clin Rheumatol* 2022; **41**: 3035–47.

16 Mease PJ, Kavanaugh A, Coates LC, *et al.* Prediction and benefits of minimal disease activity in patients with psoriatic arthritis and active skin disease in the ADEPT trial. *RMD Open* 2017; **3**: e000415.

17 van der Heijde D, Deodhar A, FitzGerald O, *et al.* 4-year results from the RAPID-PsA phase 3 randomised placebo-controlled trial of certolizumab pegol in psoriatic arthritis. *RMD Open* 2018; **4**: e000582.

18 Kavanaugh A, van der Heijde D, Beutler A, *et al.* Radiographic progression of patients with psoriatic arthritis who achieve minimal disease activity in response to golimumab therapy: Results through 5 years of a randomized, placebo-controlled study: MDA and radiographic progression in PsA patients. *Arthritis Care Res (Hoboken)* 2016; **68**: 267–74.

19 McInnes IB, Rahman P, Gottlieb AB, *et al.* Long-term efficacy and safety of guselkumab, a monoclonal antibody specific to the p19 subunit of interleukin-23, through 2 years: Results from a phase 3, randomized, double-blind, placebo-controlled study conducted in biologic-naïve patients with active psoriatic arthritis. *Arthritis Rheumatol* 2022; **74**: 475–85.

20 Coates LC, Mease PJ, Gossec L, *et al.* Minimal disease activity among active psoriatic arthritis patients treated with secukinumab: 2-year results from a multicenter, randomized, double-blind, parallel-group, placebo-controlled phase III study. *Arthritis Care Res (Hoboken)* 2018; **70**: 1529–35.

21 Coates LC, Helliwell PS. Validation of minimal disease activity criteria for psoriatic arthritis using interventional trial data. *Arthritis Care Res (Hoboken)* 2010; **62**: 965–9.

22 McInnes IB, Asahina A, Coates LC, *et al.* Bimekizumab in patients with psoriatic arthritis, naive to biologic treatment: a randomised, double-blind, placebo-controlled, phase 3 trial (BE OPTIMAL). *Lancet* 2023; **401**: 25–37.

23 Gossec L, Siebert S, Bergmans P, *et al.* Long-term effectiveness and persistence of ustekinumab and TNF inhibitors in patients with psoriatic arthritis: final 3-year results from the PsABio real-world study. *Ann Rheum Dis* 2023; **82**: 496–506.

24 Werner SG, Baraliakos X, Reckert S, *et al.* Treatment with upadacitinib in active Psoriatic Arthritis: Efficacy and safety data of the first 192 patients from the UPJOINT study, a multicentre, observational study in clinical practice. *Rheumatol Ther* 2023; **10**: 1503–18.

25 Maksabedian Hernandez EJ, Tkacz J, Lopez-Gonzalez L, Higgins K, Ogdie A, Stolshek BS.

Psoriatic arthritis treatment patterns and costs among pharmacologic treatment-naïve patients. *Am J Manag Care* 2020; **26**: e252–7.

26 Merola JF, Dennis N, Chakravarty SD, *et al.* Healthcare utilization and costs among patients with psoriasis and psoriatic arthritis in the USA-a retrospective study of claims data from 2009 to 2020. *Clin Rheumatol* 2021; **40**: 4061–70.

27 Hur P, Kim N, Dai D, Piao OW, Zheng JZ, Yi E. Healthcare cost and utilization associated with biologic treatment patterns among patients with psoriatic arthritis: Analyses from a large US claims database. *Drugs Real World Outcomes* 2021; **8**: 29–38.

28 Ogdie A, Hwang M, Veeranki P, *et al.* Health care utilization and costs associated with functional status in patients with psoriatic arthritis. *J Manag Care Spec Pharm* 2022; **28**: 997–1007.
